# Supplementation of Seaweed Extracts to the Diet Reduces Symptoms of Alzheimer’s Disease in the APPswePS1ΔE9 Mouse Model

**DOI:** 10.3390/nu16111614

**Published:** 2024-05-25

**Authors:** Nikita Martens, Na Zhan, Sammie C. Yam, Frank P. J. Leijten, Marcella Palumbo, Martien Caspers, Assia Tiane, Silvia Friedrichs, Yanlin Li, Leonie van Vark-van der Zee, Gardi Voortman, Francesca Zimetti, Dick Jaarsma, Lars Verschuren, Johan W. Jonker, Folkert Kuipers, Dieter Lütjohann, Tim Vanmierlo, Monique T. Mulder

**Affiliations:** 1 Department of Internal Medicine, Section Pharmacology and Vascular Medicine, Erasmus University Medical Center, 3015 GE Rotterdam, The Netherlandsy.li@erasmusmc.nl (Y.L.); g.voortman@erasmusmc.nl (G.V.); tim.vanmierlo@uhasselt.be (T.V.); 2 Department of Neuroscience, Biomedical Research Institute, Faculty of Medicine and Life Sciences, Hasselt University, B-3590 Hasselt, Belgium; 3 Key Laboratory of Marine Drugs, Chinese Ministry of Education, School of Medicine and Pharmacy, Ocean University of China, Qingdao 266003, China; 4 Department of Food and Drug, University of Parma, 43124 Parma, Italy; marcella.palumbo@unipr.it (M.P.);; 5 Department of Microbiology and Systems Biology, The Netherlands Organization for Applied Scientific Research (TNO), 2333 BE Leiden, The Netherlands; 6 Department Psychiatry and Neuropsychology, Division Translational Neuroscience, Mental Health and Neuroscience Institute, Maastricht University, 6200 MD Maastricht, The Netherlands; 7 Institute of Clinical Chemistry and Clinical Pharmacology, University Hospital Bonn, D-53127 Bonn, Germanydieter.luetjohann@ukbonn.de (D.L.); 8 Department of Immunology, Erasmus University Medical Center, 3000 CA Rotterdam, The Netherlands; 9 Department of Ophthalmology, Erasmus University Medical Center, 3015 GD Rotterdam, The Netherlands; 10 Department of Neuroscience, Erasmus University Medical Center, 3015 CN Rotterdam, The Netherlands; 11 Department of Pediatrics, Section of Molecular Metabolism and Nutrition, University Medical Center Groningen, University of Groningen, 9713 GZ Groningen, The Netherlands; j.w.jonker@umcg.nl (J.W.J.);; 12 European Research Institute for the Biology of Ageing (ERIBA), University Medical Center Groningen, University of Groningen, 9713 AV Groningen, The Netherlands

**Keywords:** Alzheimer’s disease, cholesterol metabolism, seaweed, oxyphytosterols, liver X receptors, inflammation

## Abstract

We previously demonstrated that diet supplementation with seaweed *Sargassum fusiforme* (*S. fusiforme*) prevented AD-related pathology in a mouse model of Alzheimer’s Disease (AD). Here, we tested a lipid extract of seaweed *Himanthalia elongata* (*H. elongata*) and a supercritical fluid (SCF) extract of *S. fusiforme* that is free of excess inorganic arsenic. Diet supplementation with *H. elongata* extract prevented cognitive deterioration in APPswePS1ΔE9 mice. Similar trends were observed for the *S. fusiforme* SCF extract. The cerebral amyloid-β plaque load remained unaffected. However, IHC analysis revealed that both extracts lowered glial markers in the brains of APPswePS1ΔE9 mice. While cerebellar cholesterol concentrations remained unaffected, both extracts increased desmosterol, an endogenous LXR agonist with anti-inflammatory properties. Both extracts increased cholesterol efflux, and particularly, *H. elongata* extract decreased the production of pro-inflammatory cytokines in LPS-stimulated THP-1-derived macrophages. Additionally, our findings suggest a reduction of AD-associated phosphorylated tau and promotion of early oligodendrocyte differentiation by *H. elongata*. RNA sequencing on the hippocampus of one-week-treated APPswePS1ΔE9 mice revealed effects of *H. elongata* on, amongst others, acetylcholine and synaptogenesis signaling pathways. In conclusion, extracts of *H. elongata* and *S. fusiforme* show potential to reduce AD-related pathology in APPswePS1ΔE9 mice. Increasing desmosterol concentrations may contribute to these effects by dampening neuroinflammation.

## 1. Introduction

Alzheimer’s Disease (AD) is the most prevalent form of dementia that is characterized by the deposition of amyloid-β (Aβ) plaques in the brain, hyperphosphorylated tau tangles, loss of synapses and neurons, and neuroinflammation, leading to progressive cognitive decline [1,2]. Over the past decades, there has been a growing interest in the role of an aberrant cholesterol metabolism in the development and progression of AD [3,4,5,6,7,8].

Liver X receptors (LXRs) α (NR1H3) and β (NR1H2) are sterol-activated nuclear receptors that control the expression of a plethora of genes involved in lipid and sterol metabolism as well as inflammatory processes [9,10,11,12,13,14]. LXRs are, therefore, considered to be promising therapeutic targets in AD. Upon the activation of LXRs by (endogenous) oxysterols, LXRs upregulate, e.g., *ABCA1* and *ABCG1*, and promote the efflux of cholesterol from astrocytes by loading endogenous ApoE to form HDL-like particles, thereby facilitating neuronal cholesterol supply and preventing the cytotoxic accumulation of intracellular lipids [15]. Furthermore, the activation of LXRs suppresses inflammation via transrepression of the NF-κB signaling pathway [16]. LXR activation was found to have protective effects in AD models [14]. Synthetic LXR agonists such as T0901317 and GW3965 were found to reduce memory decline and Aβ deposition in mouse models of AD [17,18,19,20]. Yet, their clinical application has been hampered by the concomitant induction of hepatic steatosis and hypertriglyceridemia [21,22,23].

We recently demonstrated that supplementation of the diet with seaweed-derived LXR-activating oxyphytosterol 24(*S*)-saringosterol [24], as well as the 24(*S*)-saringosterol-containing brown seaweed *S. fusiforme* [25], prevented memory decline without inducing adverse effects on hepatic lipid homeostasis. However, the relatively high amounts of toxic inorganic arsenic (iAs), specifically in *S. fusiforme*, limit its application in humans [26,27]. Here, we assess the effects of an extract of *S. fusiforme* obtained by supercritical fluid (SCF) extraction, which contains bioactive molecules but is free from iAs, as well as a lipid extract of the European brown seaweed *Himanthalia elongata* (*H. elongata*) on cognition and AD-related pathology in a mouse model for AD. Recently, we demonstrated that the *H. elongata* lipid extract, containing concentrations of 24(*S*)-saringosterol comparable to *S. fusiforme*, activates LXRs [28]. Our data demonstrated the potential of *H. elongata* extracts in the prevention of cognitive decline in APPswePS1ΔE9 mice. A similar trend was observed for the SCF extract of *S. fusiforme* but did not reach statistical significance in the current experimental design. We investigated how the extracts impacted AD-related Aβ pathology, inflammation markers, cholesterol efflux, and cholesterol metabolism to explore potential underlying mechanisms. Both extracts reduced inflammation-related markers in the brain of APPswePS1ΔE9 mice without leading to hepatic steatosis or hypertriglyceridemia. Therefore, our data suggest that diet supplementation with *H. elongata* lipid extract has the potential to prevent cognitive decline, and supplementation with either the *H. elongata* lipid extract or *S. fusiforme* SCF extract reduces inflammation-related neuropathologies of AD.

## 2. Materials and Methods

### 2.1. H. elongata and S. fusiforme Extractions

#### 2.1.1. Lipid Extracts of *H. elongata* and *S. fusiforme*

Lipids of *H. elongata* and *S. fusiforme* were extracted using the Folch method as described previously [28]. Dried *H. elongata* (The Seaweed Company, Schiedam, The Netherlands) and *S. fusiforme* (TerraSana B.V., Leimuiden, The Netherlands) were finely powdered and soaked overnight in a chloroform–methanol (2:1) mixture at room temperature and, then, exposed to Ultraviolet-C light (wavelengths: 200–280 nm). After 10 min of sonification, the chloroform–methanol mixtures were filtered with Whatman filter paper and evaporated in a rotary vacuum evaporator at 40 °C. The remaining lipid fractions were dissolved in ethanol. These ethanol extracts were used for in vitro experiments. The *S. fusiforme* lipid extract was solely used for the oligodendrocyte progenitor cells (OPCs) experiment. The *H. elongata* lipid extract contained a saringosterol concentration of 1.77 mM and a fucosterol concentration of 7.32 mM; the *S. fusiforme* lipid extract contained a saringosterol concentration of 1.12 mM and a fucosterol concentration of 6.97 mM.

#### 2.1.2. Supercritical Fluid Extraction of *S. fusiforme*

The SCF extract of *S. fusiforme* (TerraSana B.V., Leimuiden, The Netherlands) was generated by FeyeCon Development & Implementation B.V. (Weesp, The Netherlands) using a SITEC Supercritical extraction machine. The dried seaweed was milled into a powder and incubated overnight at 40 °C to remove excessive humidity. The seaweed was then introduced into the high-pressure extraction vessel, and the equipment was brought to the process conditions: 200–300 bar, 40–90 °C, and a supercritical CO_2_ (scCO_2_) flow of 4–10 kg/h. The process involved continuous recirculation of scCO_2_ that passed through the seaweed bed, moving from the bottom up within the vessel. This allowed the dissolution and extraction of seaweed constituents from the solid plant matrix into the scCO_2_. The scCO_2_ loaded with the extracted constituents was depressurized (over a controlled valve), causing CO_2_ to return to a gas state and leaving the extracted seaweed constituents (which are insoluble in the gas phase) in the separator. These sterols were collected and formed the SCF extract. The extract used for in vitro experiments contained a saringosterol concentration of 1.33 mM and a fucosterol concentration of 18.56 mM.

### 2.2. LXR Reporter Assay

#### 2.2.1. Culture of Cell Lines HEK293, CCF-STTG1, SH-SY5Y, and CHME3

Immortalized HEK293 cells (Merck, Amsterdam, The Netherlands), human astrocytoma cells (CCF-STTG1; Merck), human neuroblastoma cells (SH-SY5Y; American Type Culture Collection, Manassas, VA, USA), and human microglia cells (CHME3; a kind gift from prof. Dr. M. Tardieu, Université Paris-Sud, Bures-sur-Yvette, France) were cultured in DMEM/F-12 medium (Thermo Fisher Scientific, Waltham, MA, USA) supplemented with 10% heat-inactivated fetal calf serum (FCS; Thermo Fisher Scientific, Waltham, MA, USA) and 1% 10,000 U penicillin/10,000 μg streptomycin/mL (Thermo Fisher Scientific, Waltham, MA, USA) at 37 °C and 5% CO_2_.

#### 2.2.2. Dual Luciferase Reporter Assay

The LXR activation capacity of an SCF extract of *S. fusiforme* was assessed with a cell-based dual luciferase reporter assay as previously described [29]. A total of 1.0 × 10^6^ cells were plated in T-25 culture flasks and transfected with 1000 ng pcDNA3.1/V5H6 vector containing cDNAs for the murine LXRα or LXRβ receptors, 1000 ng of RXRα-encoding vector, and 4000 ng of LXRE-encoding vector using FuGENE^®^ 6 reagent (Promega, Leiden, The Netherlands) in culture medium (with stripped heat-inactivated FCS, Thermo Fisher Scientific) at 37 °C and 5% CO_2_. Cells transfected with 2000 ng pcDNA3.1/V5-HisA vector or 2000 ng RXRα-containing vector together with 4000 ng LXRE-containing vector served as controls. All cells were co-transfected with 1000 ng of Renilla to normalize for variations in transfection efficiency.

After transfection, cells were seeded in 96-well luminescence plates overnight and incubated for 24 h in phenol red-free DMEM/F-12 medium (Thermo Fisher Scientific) containing *S. fusiforme* SCF extract (dosage based on saringosterol concentration: 0.3 or 0.6 µM), LXRα/β agonist T0901317 (1 µM; #293754-55-9; Cayman, Ann Arbor, MI, USA), or ethanol. Cells were lysed with lysis buffer, and the luminescent Firefly and Renilla signals were detected using a Dual-Luciferase^®^ Reporter assay system (Promega) and a Victor X4 plate reader (PerkinElmer, Groningen, The Netherlands). The relative receptor activity was determined as the ratio of Firefly to Renilla luminescence, and the fold change was defined as the ratio of the relative receptor activity in cells exposed to *S. fusiforme* SCF extract or T0901317 to the relative receptor activity in ethanol-exposed cells. The experiments were performed in triplicate and were repeated three times.

### 2.3. Animals

Male APPswePS1ΔE9 (AD) mice and wildtype C57BL6/J (WT) littermates were bred through crossing of male APPswePS1ΔE9 mice (The Jackson Laboratory, Bar Harbor, ME, USA) with female C57BL6/J mice (Envigo, Horst, The Netherlands) (breeding protocol ID202132B, Hasselt University). The mice were housed in the animal facility at Hasselt University. The mice were provided with ad libitum access to food and water and were kept on a reversed 12 h light/dark cycle, with behavior experiments conducted during the dark phase. One week before the start of the behavioral experiments, the mice were individually housed. The animal procedures were approved by the ethical committee for animal experiments of Hasselt University (protocol ID202036) and was in accordance with institutional guidelines. The mice were assigned to three treatment groups using a balanced distribution approach, ensuring comparable baseline performance in cognitive tests across all groups. From 5.5 months of age and for a duration of 12 weeks, one group received standard chow (VRF1, Ssniff-Spezialdiäten GmbH, Soest, Germany) (APPswePS1ΔE9 *n* = 13, C57BL6/J *n* = 13); another group received chow supplemented with a lipid extract of *H. elongata* (192 µg/g saringosterol and 3760 µg/g fucosterol) (APPswePS1ΔE9 *n* = 13), and another group received chow supplemented with an SCF extract of *S. fusiforme* (304 µg/g saringosterol and 4797 µg/g fucosterol) (APPswePS1ΔE9 *n* = 10). The dosage of the extracts was determined based on the saringosterol content, which was similar to the dosage of saringosterol used in previous experiments involving purified 24(*S*)-saringosterol and *S. fusiforme* [24,25]. The food consumption was determined by weighing the food. The body weight of the animals was recorded on a weekly basis.

For RNA sequencing (described in Section 2.16), APPswePS1ΔE9 and WT mice received standard chow (VRF1, Sniff-Spezialdiäten GmbH, Soest, Germany) (APPswePS1ΔE9 *n* = 5, C57BL6/J *n* = 5) or chow supplemented with a lipid extract of *H. elongata* (162 µg/g saringosterol and 7393 µg/g fucosterol) (APPswePS1ΔE9 *n* = 5, C57BL6/J *n* = 5) from the age of 6 months and for a period of 1 week to evaluate treatment-specific effects. The animal procedure was approved by the ethical committee for animal experiments of Hasselt University (protocol ID202249) and were in accordance with institutional guidelines.

### 2.4. Animal Diet

#### 2.4.1. Chow Supplemented with *H. elongata* Lipid Extract

The *H. elongata* lipid extract was generated from ~7 kg of dried *H. elongata* using the Folch method as described in Section 2.1.1. The ethanol in the extract was evaporated in the vacuum rotary evaporator resulting in the final lipid extract of *H. elongata*, which was mixed through the standard chow (VRF1, Ssniff-Spezialdiäten GmbH, Soest, Germany) that was oven-dried overnight at 40 °C.

#### 2.4.2. Chow Supplemented with Supercritical Fluid Extract of *S. fusiforme*

The SCF extract of *S. fusiforme* was generated from ~70 kg of dried *S. fusiforme* by FeyeCon Development & Implementation B.V. using a SITEC Supercritical extraction machine as described in Section 2.1.2. The extract was mixed through the standard chow (VRF1, Ssniff-Spezialdiäten GmbH) that was oven-dried overnight at 40 °C.

### 2.5. Cognitive Tests

The object recognition task (ORT), object location task (OLT), and Y-maze spontaneous alternation test were performed to assess object memory, spatial memory, and spatial working memory, respectively. Prior to the baseline assessment of cognitive functioning, the mice were habituated to the arena and the objects used for cognitive testing. After baseline tests, diet supplementation with the seaweed extracts was initiated. At the end of the treatment period, the ORT, OLT, and Y-maze were performed as previously described [24,25].

#### 2.5.1. Object Recognition Task and Object Location Task

During the first trial of the ORT and OLT, the mice were exposed to two similar objects for 4 min after which they were placed back in their home cage. After an inter-trial interval of 2.5 h (ORT) or 5 h (OLT), a second trial of 4 min was performed. During this second trial, the mice were exposed to either one familiar object from trial 1 and one novel object (ORT) or to the two objects from trial 1 of which one was displaced (OLT). The times the mice spent exploring each object during trial 1 and trial 2 were recorded manually by two researchers who were blinded to the experimental groups. As a measure of object memory (ORT) and spatial memory (OLT), a discrimination index (D2) for trial 2 was calculated as follows: (exploration time for novel [ORT] or displaced [OLT] object) − (exploration time for familiar [ORT] or stationary [OLT] object)/(total exploration time in trial 2). A D2 value above 0 indicated a preference for the novel/displaced object. The arena and study objects were thoroughly cleaned with 70% ethanol prior to each trial to prevent olfactory cues. Data of animals that did not reach the minimum of 4 s of exploration in trial 1 or trial 2 and extreme values, as determined by Dixon’s exclusion principles [30,31], were excluded from further analysis.

#### 2.5.2. Y-Maze Spontaneous Alternation Test

The Y-maze spontaneous alternation test was conducted with a maze consisting of three arms of equal length, separated at an angle of 120°. The mice were placed in one of the arms and explored the maze for 2 min. The arm entries were recorded manually. An arm entry requires that all four limbs of the mouse are within the arm. The spatial working memory was assessed by defining the percentage of alternations as follows: number of alternations/(total number of entries − 2) × 100. The number of alternations was defined as subsequently entering the three different arms. A percentage of alternation of above 50% was considered an indication of a well-functioning working memory. The Y-maze was thoroughly cleaned with 70% ethanol prior to each trial to prevent olfactory cues. Extreme values, as determined by Dixon’s exclusion principles [30,31], were excluded from further analysis.

After the memory assessment, the mice were sacrificed to obtain tissues for further analysis.

### 2.6. Tissue sample Preparation

The mice were anesthetized via intraperitoneal injection of Dolethal (200 mg per kg body weight; Vetoquinol, Aartselaar, Belgium). Blood was collected via cardiac puncture and centrifuged at 4000× *g* for 5 min to obtain the serum. The obtained serum was stored at −80 °C until further use. The blood collection was followed by transcardiac perfusion with Heparin-phosphate-buffered saline (PBS). Brains were isolated and divided into the cerebellum and the remaining two hemispheres. The left hemisphere was fixed in formalin and embedded in paraffin for immunohistochemistry (IHC). The cerebellum, cortex, and forebrain from the right hemisphere were snap-frozen and cryopreserved for sterol analysis, Aβ_42_ analyses, and p-tau quantification, respectively. The liver was isolated, snap-frozen, and stored at −80 °C for sterol and triglyceride measurements and for quantitative Real-Time PCR (Q-PCR). A part of the left liver lobe was embedded in an OCT mounting medium (VWR International, Leuven, Belgium) for cryotomy and subsequent staining of neutral lipids.

### 2.7. Triglyceride and Neutral Lipid Quantification

Lipid extraction from liver homogenates was performed as described by Bligh and Dyer [32]. The triglyceride concentrations in hepatic lipid extracts and serum were determined with enzymatic reagent kits according to the manufacturer’s instructions (DiaSys Diagnostic Systems, Holzheim, Germany).

Hepatic neutral lipids were quantified in liver sections stained with Oil Red O, as described previously [24]. Tissue Tek-embedded livers were cut with a cryostat CM3050S (Leica, Wetzlar, Germany). The sections (12 µm thick) were mounted on SuperFrost Plus adhesion slides (Thermo Fisher Scientific, Waltham, USA) and stored at −20 °C until use. These liver sections were fixed in 4% neutral buffered formalin, washed with tap water, and rinsed with 60% isopropanol. Hepatic lipids were stained with Oil Red O (Polysciences Inc., Warrington, FL, USA) for 15 min after which the liver sections were rinsed with 60% isopropanol and covered with a coverslip. Digital images of the sections were obtained using a Leica DMLB microscope (Leica Microsystems, Rijswijk, The Netherlands) equipped with software from the Leica Applications Suite (Leica Microsystems, Rijswijk, The Netherlands). The surface area of the Oil Red O staining was quantified using Fiji ImageJ 1.53t software by defining the pixel intensity of the staining.

### 2.8. RNA Isolation and cDNA Synthesis

Tissue samples were homogenized using the BioSpec Mini-Beadbeater (Biospec Products, Bartlesville, OK, USA). RNA was extracted from these tissue homogenates using Trizol (Thermo Fisher Scientific) and converted into cDNA through reverse transcription using the Maxima H Minus First Strand cDNA Synthesis Kit with dsDNase (Thermo Fisher Scientific), following the manufacturer’s instructions. The RNA quality was assessed using the RNA Nano chip on the Agilent 2100 Bioanalyzer (Agilent Technologies, Amstelveen, The Netherlands).

### 2.9. Quantitative Real-Time PCR

Q-PCR was carried out using 10 ng cDNA on a CFX384 Thermal Cycler (Bio-Rad laboratories with the PowerTrack^TM^ SYBR Green Master Mix (Applied Biosystems) under the following cycling conditions: 95 °C for 2 min and 40 cycles of [95 °C for 15 s, 60 °C for 60 s]. The intron-spanning primers (Table 1) were designed with Primer-BLAST. The relative quantification of gene expression was achieved with the comparative Ct method. The data were normalized to stable reference genes (*ACTB*, *B2M*, *HPRT1*, and *SDHA*) and expressed as fold changes relative to the vehicle-treated WT mice.

### 2.10. Lipoprotein Profile

The cholesterol concentrations in very-low-density lipoproteins (VLDLs), low-density lipoproteins (LDLs), and high-density lipoproteins (HDLs) in serum were determined using fast protein liquid chromatography (FPLC), as previously described [33,34]. The system consisted of a PU4180LPG quaternary pump with an LG-980-02 linear degasser, FP920 fluorescence, and UV4075 UV/VIS detectors (Jasco, Tokyo, Japan). After injection of 25 µL of serum (1:1 diluted in TBS (pH 7.4)), the lipoproteins were separated utilizing a Superose 6 HR 10/30 column (GE Healthcare Hoevelaken, The Netherlands) that operated at a flow rate of 0.31 mL/minute. For cholesterol detection, an additional R-PU-4081i Right extension pump (Jasco, Tokyo, Japan) was used for introducing cholesterol PAP enzymatic reagent (Diasys, Holzheim, Germany) at a flow rate of 0.1 mL/minute. The ChromNav chromatographic software, version 2.0 (Jasco, Tokyo, Japan) was used for analysis of the chromatogram results. For quantitative analysis of the separated lipoproteins, commercially available lipid plasma standards (low, medium, and high; SKZL, Nijmegen, The Netherlands) were employed.

### 2.11. Immunohistochemistry

#### 2.11.1. Brain—Paraffin Sections

The left hemispheres of the brain were used for IHC. First, the left hemispheres were fixed in 10% formalin and embedded in paraffin using a Thermo Scientific Excelsior ES Tissue Processor (Thermo Fisher Scientific, Waltham, MA, USA). This process involved incubating the hemispheres in increasing concentrations of ethanol (70% for 1 h, 80% for 1 h, 95% for 1 h, 100% for 1.5 h), followed by xylene for 1.5 h, and paraffin wax at 60 °C for 2 h. The embedded hemispheres were subsequently cut with an HM 330 Motorized Rotary Microtome (Microm GmbH., Heidelberg, Germany) to obtain 5 μm sections. The obtained sections were mounted on glass slides, air-dried overnight at 37 °C, and stored at room temperature (RT) until further use.

For immunohistochemical staining, the sections were first deparaffinized by incubating them in xylene for 10 min, followed by decreasing concentrations of ethanol (100% for 6 min, 96% for 3 min, and 70% for 3 min). After washing the sections for 10 min in Tris-buffered saline (TBS)/0.3% Triton X-100, the sections were incubated in 10 mM citrate buffer (pH 6.0) for 10 min at 100 °C. Once cooled down, the sections were rinsed in PBS, incubated for 10 min at RT in 3% H_2_O_2_ diluted in methanol, and rinsed for 5 min with TBS/0.3% Triton X-100. The sections were incubated with blocking solution (5 *v*/*v*% bovine serum albumin in TBS/0.3% Triton X-100) for 1 h at RT before adding 150 μL of the primary antibody (Aβ: clone 3D6 mouse anti-human (1:8000); Iba1: Cat.#019-19741, Wako Chemicals USA, Inc., Richmond, VA, USA (1:1000); CD68: MCA1957, Bio-Rad Laboratories Inc., Hercules, CA, USA (1:100); GFAP: Dako-Z0334, Agilent Technologies, Glostrup, Denmark (1:2000)) in blocking solution. The Aβ antibody (clone 3D6) was provided by P. Martinez-Martinez and M.R. Losen (Mental Health and Neuroscience Institute, Maastricht University, Maastricht, The Netherlands). After an overnight incubation with the primary antibody, the slides were then incubated overnight at 4 °C. After washing the sections five times with TBS/0.3% Trition X-100, the sections were incubated with 150 μL of the appropriate biotinylated secondary antibodies (1:500) for 30 min at RT. After rinsing with TBS/0.3% Triton X-100, 150 μL avidin-biotin complex reagent (ABC kit, Vector Laboratories, Newark, CA, USA) was added to each section and incubated for 30 min at RT. The avidin-biotin complex reagent was washed away. Next, diaminobenzidine (DAB; ImmPACT DAB, Vector Laboratories, Newark, CA, USA) was added to each section and, as soon as the sections developed color, the slides were dipped in distilled water (dH_2_O). Only for the Aβ staining, the slides were counterstained with Mayer Hematoxylin for 30 s and washed in dH_2_0 for 10 min. Subsequently, all sections were dehydrated by incubating the sections in 70% ethanol for 1 min, 100% ethanol for 1 min, and xylene for 1 min. The coverslips were mounted with Entallan (Sigma-Aldrich, St. Louis, MO, USA).

#### 2.11.2. Liver—Cryosections

Tissue Tek-embedded livers were cut as described in Section 2.7 and stored at −20 °C until use. The liver sections were fixated for 10 min in acetone after which the sections were rehydrated for 10 min and washed in PBS. The sections were subsequently incubated in 0.3% H_2_O_2_ for 5 min and washed in PBS. After incubation for 30 min in blocking solution (PBS containing 1% bovine serum albumin (BSA) and 10% normal goat serum), the sections were incubated for 1 h with primary F4/80 antibody MCA497G (provided by K. Wouters (Cardiovascular Research Institute Maastricht (CARIM), Maastricht University, Maastricht, The Netherlands)) in PBS-1% BSA (1:200). After washing with PBS, the sections were incubated with secondary horseradish peroxidase (HRP)-labeled antibody (1:100) in PBS-1% BSA for 1 h. The sections were washed with PBS and incubated with DAB (ImmPACT DAB, Vector Laboratories, Newark, CA, USA). After 5 min, the sections were dipped in dH_2_O, and coverslips were mounted with Kaiser’s glycerol/gelatin.

#### 2.11.3. Image Analysis

Images of the sections were acquired using a Nanozoomer 2.0 HT slide scanner (Hamamatsu Photonics K.K., Shizuoka, Japan). The surface areas of Iba1, CD68, GFAP, and F4/80 staining were determined using Trainable Weka Segmentation in ImageJ [35]. The surface area of the Aβ staining was quantified using Fiji ImageJ software by defining the pixel intensity of the staining. The Iba1-positive somata were manually counted.

### 2.12. Quantification of Extracellular Soluble Aβ42

To quantify Aβ_42_ concentrations using ELISA, the cortex of the right hemisphere of the brains from APPswePS1ΔE9 mice was homogenized in TBS/0.1% Triton X-100 containing 2% complete protease inhibitor cocktail (pH 7.2; Roche Diagnostics Ltd., Mannheim, Germany) and centrifuged at 21,000× *g* for 20 min at 4 °C. The supernatant with the extracellular soluble Aβ fraction was collected and stored at −80 °C until further use. Aβ_42_ concentrations in the samples were quantified using an Aβ_42_ ELISA kit (Invitrogen, Carlsbad, CA, USA) according to the manufacturer’s instructions. The Aβ_42_ concentrations were normalized to the total protein concentrations in the samples loaded, which were determined via a BCA protein assay kit (Thermo Fischer Scientific, Waltham, MA, USA) per the manufacturer’s instructions.

### 2.13. Cytokine Measurements

#### 2.13.1. THP-1 Cell Culture

Human monocytic cell line THP-1 cells were kindly provided as a gift by Conny Van Holten-Leelen (Department of Immunology, Erasmus MC, Rotterdam, The Netherlands). THP-1 cells were cultured in suspension at 37 °C and 5% CO_2_ using RPMI Medium 1640 + GlutaMAX-I + 25 mM HEPES (Thermo Fisher Scientific, Waltham, MA, USA) supplemented with 10% heat-inactivated FCS (Thermo Fisher Scientific), 1% 10,000 U penicillin/10,000 μg streptomycin/mL (Thermo Fisher Scientific), 1 mM sodium pyruvate (Thermo Fisher Scientific), 0.05 mM 2-mercaptoethanol (Thermo Fisher Scientific), and 2.5 mg/mL glucose (Merck, St. Louis, MO, USA), hereafter referred to as culture medium. THP-1 cells were seeded in a 24-well plate at a density of 0.5 × 10^6^ cells per well per 600 µL and differentiated into macrophages using culture medium supplemented with 100 ng/mL Phorbol 12-myristate 13-acetate (PMA; Merck, St. Louis, MO, USA). After 72 h, unattached cells were removed, and the attached cells were further cultured for 24 h using 300 µL culture medium per well supplemented with 50 ng/mL PMA to obtain THP-1-derived macrophages. These macrophages were refreshed with 500 µL culture medium without PMA but with 10 ng/mL of LPS (Merck, St. Louis, MO, USA) to initiate inflammation. To test the anti-inflammatory function of *H. elongata* lipid extract and *S. fusiforme* SCF extract, these extracts, together with LPS, were added to the cells and incubated at 37 °C and 5% CO_2_ for 24 h. The extracts were added based on their saringosterol concentration, with a final saringosterol concentration of 0.6 µM in the medium. Cell culture supernatants were harvested and stored at −80 °C until further use.

#### 2.13.2. Cytokines Measurements

Pro- and anti-inflammatory cytokines including IL-1β, IL-8, TNFα, IL-6, and IL-10 in the culture supernatants were measured using Human Magnetic Luminex Discovery Assay Kit (Kit Lot number: L140180, Cat.# LXSAHM, R&D systems, Abingdon, UK) according to manufacturer’s instruction. Data were acquired using BioPlex MAGPIXTM Multiplex Reader and analyzed using Bio-Plex Manager MP software (Bio-Rad, Hercules, CA, USA).

### 2.14. Cholesterol Efflux

#### 2.14.1. THP-1 Cell Culture

The human monocytic THP-1 cells (European Collection of Authenticated Cell Cultures, purchased from Sigma-Aldrich, St. Louis, MO, USA) were cultured in RPMI 1640 medium (Euroclone, Milan, Italy) supplemented with HEPES, 0.05 mM 2-Mercaptoethanol, 0.5% *v*/*v* gentamicin, 2.5 mg/mL glucose, 1 mM sodium pyruvate (all from Thermo Fisher Scientific, Waltham, MA, USA), and 10% FCS (Euroclone, Milan, Italy). Cells were seeded in 24-well plates (500,000 cells/well) and incubated for 72 h in the presence of 100 ng/mL phorbol 12-myristate 13-acetate (PMA; Sigma-Aldrich, St. Louis, MO, USA) to allow differentiation in macrophages.

#### 2.14.2. Cholesterol Efflux Assay

Cellular cholesterol efflux was assessed in THP-1-derived macrophages using a standardized radio-isotopic technique [36]. In detail, cells were labeled with [1,2-^3^H(N)]-Cholesterol (PerkinElmer, Waltham, MA, USA) at 2 μCi/mL in the presence of 1% FCS-containing RPMI and 2 μg/mL Sandoz 58-035 (Sigma-Aldrich, St. Louis, MO, USA), an acyl-coenzyme A: cholesterol acyltransferase (ACAT) inhibitor preventing cholesteryl ester accumulation. Subsequently, cell monolayers were equilibrated for 20 h in 0.2% BSA (Sigma-Aldrich, St. Louis, MO, USA)-containing medium supplemented with ethanol, the LXR/RXR agonists 22-hydroxycholesterol (22-OHC)/9-cis-Retinoic acid (9cRA) (5 µg/mL of 22OH-C and 10 µM of 9cRA; both from Sigma-Aldrich, St. Louis, MO, USA), and increasing concentrations of *H. elongata* lipid extract or *S. fusiforme* SCF extract, in the presence of the ACAT inhibitor. The extracts were added based on saringosterol concentration, with a final saringosterol concentration of 0.3, 0.6, 1.25, or 2.5 µM for *H. elongata* and a final saringosterol concentration of 0.15, 0.3, 0.6, or 1.25 µM for *S. fusiforme* SCF (concentration ranges tolerated by cells). Cholesterol efflux was promoted by a 6 h incubation with 10 µg/mL of lipid-free human apolipoprotein A-I (ApoA-I; Sigma-Aldrich, St. Louis, MO, USA) or 12.5 µg/mL of human high-density lipoprotein (HDL; Sigma-Aldrich, St. Louis, MO, USA). Cholesterol efflux results were expressed as a percentage of radiolabeled cholesterol released into the medium relative to the total radioactivity incorporated by the cells [37].

### 2.15. OPC Differentiation

#### 2.15.1. Primary OPC Culture

Primary OPCs were isolated from postnatal day 0 C57BL/6J mouse pups, as described previously [38]. In summary, cortices were isolated, meninges were removed, and cells were dissociated through a 20 min incubation in a papain-DNAse solution (3 U/mL; Sigma-Aldrich) at 37 °C. The resulting mixed glial cell suspension was cultured in poly-L-lysine (5 µg/mL)-coated T75 culture flasks, with DMEM high glucose medium (Sigma-Aldrich), supplemented with 100 U/mL penicillin, 100 µg/mL streptomycin (Invitrogen), and 10% heat-inactivated FCS (Biowest, Nuaillé, France). The cells were maintained at 37 °C in a humidified atmosphere with 8.5% CO_2_. On day 14, OPCs were obtained after an overnight shake-off at 250 rpm and 37 °C and, then, seeded (1.5 × 10^5^ cells/cm^2^) in differentiation medium. Subsequently, OPCs were treated with *S. fusiforme* or *H. elongata* lipid extracts for six days, receiving a treatment boost every 48 h. The extracts were added based on their saringosterol concentration, with a final saringosterol concentration of 0.6 µM in the medium.

#### 2.15.2. Immunocytochemistry

Primary OPCs were fixed at room temperature for 30 min using 4% paraformaldehyde (PFA). Following fixation, cells were subjected to blocking and permeabilization with 0.1% Triton X-100 in 1% BSA for 30 min. This was followed by a 4 h incubation with a mouse anti-O4 IgM (1:1000; R&D Systems) antibody at room temperature. Next, cells were incubated with an Alexa fluor secondary antibody (Invitrogen) for one hour, and nuclei were counterstained with 1 µg/mL DAPI (Sigma). Finally, coverslips were mounted using Fluoromount-G Mounting Medium (Invitrogen) and subjected to analysis using a Leica DM2000 LED fluorescence microscope. Quantitative analysis of images was conducted using Fiji, ImageJ software.

### 2.16. RNA Sequencing and Data Analysis

We analyzed the transcriptome of hippocampus samples of APPswePS1ΔE9 and WT mice treated with *H. elongata* extract for one week (the animal study was conducted as described in Section 2.3). The hippocampus of the left brain hemisphere of these animals was homogenized, and RNA was extracted as described in Section 2.8. RNA integrity was examined utilizing the RNA 6000 nano Lab-on-a-Chip kit and the bioanalyzer 2100 (Agilent Technologies, Amstelveen, The Netherlands). The NEBNext Ultra II Directional RNA Library Prep Kit (NEB #E7760S/L, New England Biolabs, Ipswich, MA, USA) was utilized to process the samples. Briefly, mRNA was isolated from total RNA using the oligo-dT magnetic beads. After fragmentation of the mRNA, cDNA was synthesized, ligated with the sequencing adapters, and amplified by PCR. The quality and yield of the amplicon were measured with a Fragment Analyzer (Agilent Technologies, Amstelveen, The Netherlands). The size of the resulting product was consistent with the expected size distribution (a broad peak between 300 and 500 bp). Clustering and DNA sequencing, using the Illumina NovaSeq6000, were performed according to the manufacturer’s protocols of service provider GenomeScan B.V. (Leiden, The Netherlands), using a concentration of 1.1 nM of amplicon library DNA and yielding at least 15 million sequencing clusters per sample and 150 nt paired-end reads. The genome reference and annotation file Mus_musculus. GRCm38.gencode.vM19 were used for analysis in the FastA and GTF format. The reads were aligned to the reference sequence using the STAR 2.5 algorithm with default settings (https://github.com/alexdobin/STAR, accessed on 26 September 2023). Based on the mapped read locations and the gene annotation, HTSeq-count version 0.6.1p1 was used to count how often a read was mapped on the transcript region. These counts served as input for statistical analysis using the DEseq2 package [39]. Data analysis was performed by performing a pathway enrichment analysis using selected differentially expressed genes (*p*-value < 0.01) which were used as input in an Ingenuity Pathway Analysis (IPA, www.ingenuity.com, accessed on 9 October 2024). Pathways with *p*-values  <  0.05 were considered to be significant.

### 2.17. Sterol Measurement

The sterol concentrations in cells, cerebellum, liver, and serum were determined by gas chromatography–mass spectrometry (GC-MS) as described previously [40]. Briefly, the cell and tissue samples were first spun in a speed vacuum dryer to relate individual sterol concentrations to dry weight. Sterols were extracted from the dried tissues with a 5 mL mixture of chloroform–methanol, after which 1 mL of sterol extracts was evaporated to dryness. Subsequently, 1 mL of distilled water was added to the samples. To extract the neutral sterols, 3 mL of cyclohexane was added twice. The combined cyclohexane phases were again evaporated to dryness under a stream of nitrogen at 63 °C, and the sterols were dissolved in 100 μL n-decane. After transfer to GC-vials, the sterols were converted to trimethylsilyl ethers and incubated at 60 °C for 1 h. Cholesterol concentrations were determined in a gas-chromatograph-flame ionization detector (GC-FID) with 50 μL 5α-cholestane-solution (1 mg/mL 5α-cholestane in cyclohexane) as an internal standard. Concentrations of plant sterols (saringosterol, fucosterol, avenasterol, brassicasterol, campesterol, campestanol, sitosterol, sitostanol, and stigmasterol), cholesterol precursors (lanosterol, lathosterol, and desmosterol), and cholesterol metabolites (24-hydroxycholesterol (24-OHC), 27-hydroxycholesterol (27-OHC), 7α-hydroxycholesterol (7α-OHC), and cholestanol) were determined using GC-MS, with 10 μL epicoprostanol (100 μg/mL in cyclohexane) as an internal standard.

### 2.18. Tau and p-Tau Measurements in Differentiated SH-SY5Y Cells

#### 2.18.1. SH-SY5Y Cell Culture

For tau analysis, SH-SY5Y cells (passage 15–25; CRL-2266; American Type Culture Collection, USA) were cultured in DMEM/F12 medium with GlutaMAX^TM^ (Cat.#10565018, Thermo Fisher Scientific) supplemented with 10% heat-inactivated FCS (Cat.#26140079 Thermo Fisher Scientific) and 1% penicillin/streptomycin (Cat.#15140-122, Gibco, Grand Island, NY, USA). The cell medium was refreshed every 2 or 3 days, and cells were passed when they attained 80–95% confluence. For the differentiation of SH-SY5Y cells into neuron-like cells, 2.5 × 10^5^ cells were seeded in T-25 flasks. After 24 h, the maintenance medium was replaced by DMEM/F12 (1:1) medium with GlutaMAX™ supplemented with 1% FCS, 1% penicillin/streptomycin, and 10 μM retinoic acid (RA; Cat.#R2625, Sigma-Aldrich). The differentiation medium was refreshed every other day for a total of 5 days. After these 5 days, the medium was replaced with Neurobasal™ Medium (Cat.#11570556, Thermo Fisher Scientific) supplemented with 50 ng/mL BDNF (Cat.#ab206642, Abcam, Waltham, MA, USA), B-27 (Cat.#11530536, Thermo Fisher Scientific; 1:50), 20 mM KCI, 2 mM Glutamax^TM^, 2 mM dibutryryl cyclic AMP (Cat.#SC-201567, Bio-Connect BV, Huissen, The Netherlands), 10 μM RA, and 1% penicillin/streptomycin. This medium was refreshed every other day for a duration of 5 days, followed by 24 h of incubation with *H. elongata* lipid extract, *S. fusiforme* SCF extract, saringosterol (1.25, 2.5, or 5.0 µM; COMFiON BV, Leimuiden, The Netherlands), desmosterol (1.25, 2.5, or 5.0 µM; Cat.#700060P, Avanti, Tonawanda, NY, USA), LXR agonist T0901317 (1 µM; Cat.#293754-55-9; Cayman, Ann Arbor, MI, USA), or ethanol. The extracts were added based on saringosterol concentration, with a final saringosterol concentration of 0.6, 1.2, 2.4, or 4.8 µM for *H. elongata* and a final saringosterol concentration of 0.075, 0.15, 0.3, or 0.6 µM for *S. fusiforme* SCF. The cells were subsequently lysed with 150 µL cold Radioimmunoprecipitation assay (RIPA) lysis and extraction buffer (Cat.#89900, Thermo Fisher Scientific) supplemented with protease inhibitors (Cat.#P0044 Sigma-Aldrich; 1:100) and phosphatase inhibitors (Cat.# 11697498001, Roche; 0.4 mg/mL). The cells were then harvested by scraping and directly placed on ice. After centrifugation at 14,000× *g* for 30 min at 4 °C, the supernatant was collected and stored at −80 °C until further use.

#### 2.18.2. Protein Isolation from the Forebrain

The forebrains were homogenized using the BioSpec Mini-Beadbeater (Biospec Products, Bartlesville, OK, USA) in cold RIPA buffer supplemented with protease inhibitors (Cat.#P0044 Sigma-Aldrich; 1:100). After the homogenates were centrifuged (30 min, 14,000× *g*, 4 °C), the supernatant was collected and stored at −80 °C until further use.

#### 2.18.3. Western Blot for TAU-5 and AT-8 Measurements

The protein concentrations in the cell and forebrain isolations were determined using a BCA protein assay kit (Thermo Fischer Scientific), per the manufacturer’s instructions. An amount of 10–20 µg of the protein sample was dissolved in Laemmli sample buffer (Cat.#161-0747, Bio-Rad; 1:4) and equalized with RIPA buffer. Subsequently, the samples were incubated at 100 °C for 3 min and centrifuged. The protein-containing supernatant (15 µg protein) was loaded onto a 4–20% Mini-PROTEAN^®^ TGX™ Precast Gel (Cat.#4561096, Bio-Rad). After gel electrophoreses of 15 min at 100 V and 1–2 h at 200 V, the gels were transferred onto a polyvinylidene difluoride (PDVF) membrane (Cat.#1704157, Bio-Rad) using a Trans-Blot^®^ Turbo™ Transfer System (Cat.#1704150, Bio-Rad).

The membranes were washed in 1×TBS and incubated in blocking buffer (5% skim milk in TBS containing 0.1% Tween (TBST) for GAPDH and TAU-5 or 5% BSA in TBST for AT8) for 30 min, after which the membranes were incubated overnight with the primary antibody (1:1000 TAU-5 (Cat.#AHB0042, Thermo Fisher Scientific); 1:200 AT8 (Phospho-Tau (Ser202, Thr205), Cat.#MN1020, Thermo Fisher Scientific); 1:2000 GAPDH (Cat.#GT239, Thermo Fisher Scientific)) at 4 °C. After the overnight incubation with primary antibody, the membranes were washed with TBST and incubated with the secondary antibody (1:4000 goat anti-mouse (Cat.#179-1019, Bio-Rad) for TAU-5 and AT-8 or 1:4000 goat-anti rabbit (Cat.#170-6516, Bio-Rad) for GAPDH). The protein bands were visualized by incubating the membranes with enhanced chemiluminescence substrate (ECL, Cat.#A38555, Thermo Fisher Scientific) for 1 min, and images were obtained with an Amersham Imager 600. The quantification was performed in ImageJ, by determination of the surface area and intensity of the protein bands. The data were normalized to the reference protein GAPDH and expressed as fold change relative to ethanol-incubated cells.

### 2.19. Statistical Analyses

The statistical analyses were conducted with PRISM GraphPad 8. The data were analytically tested for normal distribution with the Shapiro–Wilk normality test and visually checked by plotting the data in a histogram. For data showing a normal distribution, we performed an unpaired *t*-test for comparison of vehicle-treated WT and APPswePS1ΔE9 mice, or we performed an ordinary one-way ANOVA (with Dunnett’s multiple comparisons test) for analysis of the treatment effect in APPswePS1ΔE9 mice. For not-normally distributed data, we performed a Mann–Whitney U test for comparison of vehicle-treated WT and APPswePS1ΔE9 mice or a Kruskal–Wallis test (with Dunn’s multiple comparisons test) for analysis of the treatment effect in APPswePS1ΔE9 mice. The behavioral data were analyzed with a one-sample *t*-test [41]. Significances between vehicle-treated WT and APPswePS1ΔE9 mice are presented as # *p* < 0.05, ## *p* < 0.01, and ### *p* < 0.001; significances between vehicle-treated and extract-treated APPswePS1ΔE9 mice are presented as * *p* < 0.05, ** *p* < 0.01, and *** *p* < 0.001.

## 3. Results

### 3.1. The Supercritical Fluid Extract of S. fusiforme Activated LXRα and LXRβ

**Figure 1:**
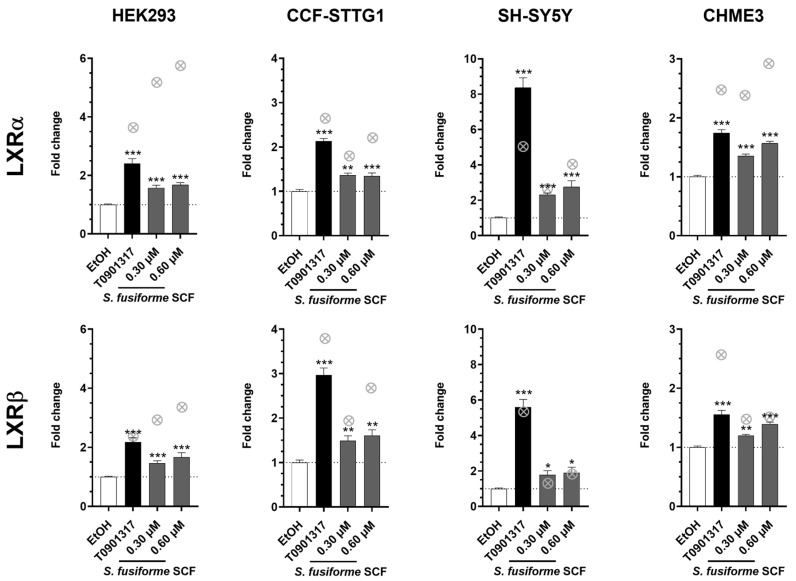
The *S. fusiforme* SCF extract activates LXRα and LXRβ. The *S. fusiforme* SCF extract was tested for its LXRα- and LXRβ-activating capacity in HEK293, CCF-STTG1, SH-SY5Y, and CHME3 cells. Cells were incubated with the extract—which was added based on its saringosterol concentration (on the *X*-axis)—or with T0901317 (1 µM) as a positive control. The data are expressed as fold change relative to EtOH and presented as mean ± SEM of three independent experiments performed in triplicate (*n* = 9). Data were analyzed with one-way ANOVA (Kruskal–Wallis test with Dunn’s multiple comparisons test). Fold changes relative to the EtOH control: * *p* < 0.05, ** *p* < 0.01, *** *p* < 0.001. ⊗: Data on LXR activation by *H. elongata* lipid extract from Ref. [28].

We first tested whether *S. fusiforme* SCF extract activated LXRs, as previously demonstrated with the chloroform–methanol extracts of *S. fusiforme* and *H. elongata* [25,28], using a cell-based dual luciferase reporter assay. The data demonstrated that the *S. fusiforme* SCF extract activated both LXRα and LXRβ in HEK293, CCF-STTG1, SH-SY5Y, and CHME3 cells (Figure 1).

### 3.2. Food Intake, Body Weight, and Lipid Homeostasis in Liver and Serum upon Supplementation of H. elongata Lipid Extract and S. fusiforme SCF Extract to the Diet

Next, we mixed the *H. elongata* lipid extract and *S. fusiforme* SCF extract into the diet of APPswePS1ΔE9 mice for 12 weeks to examine their effects on cognitive performance and the development of AD-related neuropathology.

**Figure 2:**
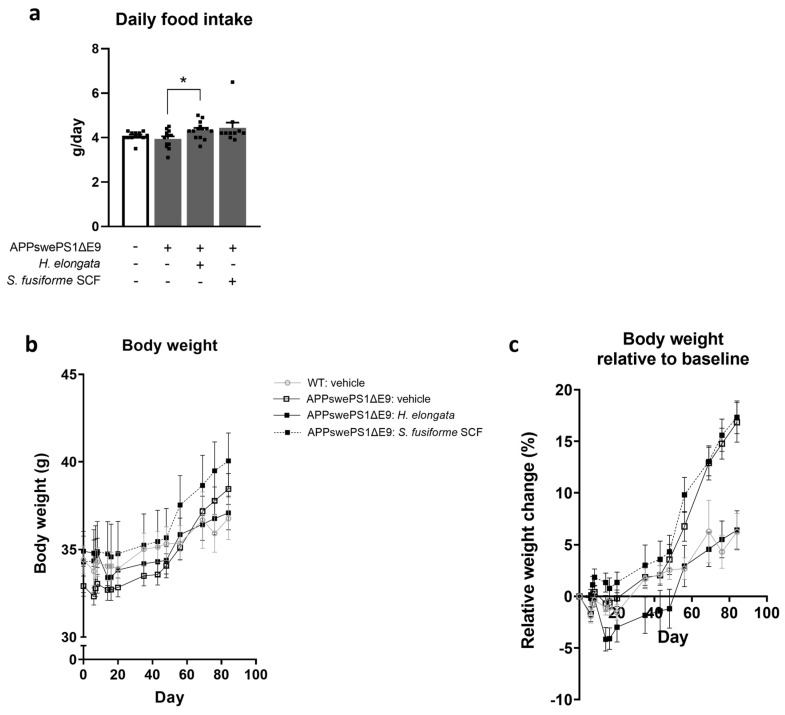
Food intake and body weight upon supplementation of *H. elongata* lipid extract and *S. fusiforme* SCF extract to the diet. The daily food intake (**a**), body weight (**b**), and body weight relative to baseline (**c**) are presented (*n* = 10–14 per group). Data are presented as mean ± SEM. Differences between vehicle-treated APPswePS1ΔE9 and WT mice were analyzed with a Mann–Whitney U test (no statistically significant differences detected); Treatment effects in APPswePS1ΔE9 mice were analyzed with a Kruskal–Wallis test (with Dunnett’s/Dunn’s multiple comparisons test) (* *p* < 0.05).

Although the food intake of vehicle-treated WT and APPswePS1ΔE9 mice did not differ (*p* = 0.5871) (Figure 2a), APPswePS1ΔE9 mice gained more weight during the 12 weeks of the experiment than WT mice did (*p* = 0.0008) (Figure 2b,c). The serum and liver triglyceride concentrations were not different in APPswePS1ΔE9 mice compared to WT mice (*p* = 0.2351 and *p* = 0.1619, respectively) (Figure S1a,b). However, vehicle-treated APPswePS1ΔE9 mice demonstrated an elevated hepatic neutral lipid content compared to WT mice (*p* = 0.0244) (Figure S1c).

APPswePS1ΔE9 mice receiving a diet supplemented with *H. elongata* extract demonstrated a slightly higher food intake (*p* = 0.0323) (Figure 2a). Nevertheless, the APPswePS1ΔE9 mice that received an *H. elongata*-supplemented diet gained less weight throughout the experiment than vehicle-treated mice (*p* = 0.0022) (Figure 2b,c). Diet supplementation with the *S. fusiforme* SCF extract did not significantly affect the food intake (*p* = 0.2209) (Figure 2a) or weight gain of APPswePS1ΔE9 mice during the experiment (*p* > 0.9999) (Figure 2b,c). The extracts tended to reduce the serum triglyceride concentration in APPswePS1ΔE9 mice (ANOVA: *p* = 0.0625) (Figure S1a). The hepatic triglyceride concentration and the hepatic neutral lipid content were not significantly affected by the *H. elongata* lipid extract or by the *S. fusiforme* SCF extract (Figure S1b,c).

Diet supplementation with the *H. elongata* and *S. fusiforme* SCF extract both decreased the expression of *SREBF1* in the liver (*p* = 0.0385 and *p* = 0.0420, respectively) (Figure S1d) but did not significantly affect the expression of the SREBF1 target genes *FASN*, *ACACA*, and *SCD1* (Figure S1e–g). The *S. fusiforme* SCF extract decreased the relative levels of VLDL-c (*p* = 0.0021) and increased the relative LDL-c (*p* = 0.0225) (Figure S1h,i). No effect of *H. elongata* supplementation was detected on relative VLDL-c, LDL-c, and HDL-c levels in serum (Figure S1h–j).

### 3.3. Prevention of Cognitive Decline in APPswePS1ΔE9 Mice

**Figure 3:**
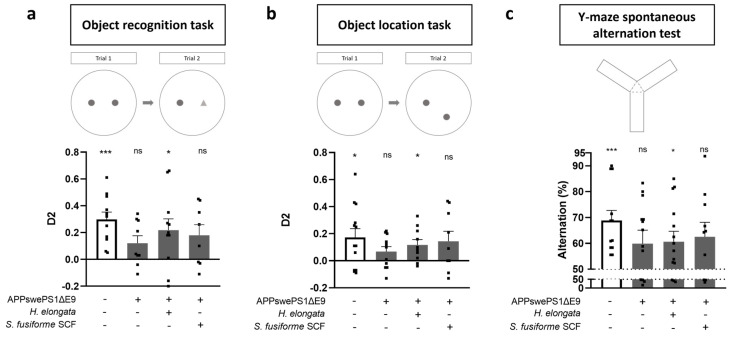
Diet supplementation with a lipid extract of *H. elongata* prevents cognitive decline in APPswePS1ΔE9 mice. The cognitive performance of WT and APPswePS1ΔE9 mice that received *H. elongata* extract or *S. fusiforme* SCF extract was determined with an object recognition task (ORT) (**a**), object location task (OLT) (**b**), and Y-maze spontaneous alternation test (**c**). Data are represented as mean ± SEM (*n* = 8–14 per group) and were analyzed with a one-sample *t*-test. D2 values relative to 0: * *p* < 0.05, *** *p* < 0.001, ns: *p* ≥ 0.05.

At baseline, both APPswePS1ΔE9 and WT mice demonstrated functional spatial memory (Figure S2). After 12 weeks of supplementing the extracts, APPswePS1ΔE9 and WT mice were examined for their cognitive performance in the ORT (Figure 3a), OLT (Figure 3b), and Y-maze (Figure 3c). Consistent with previous reports [24,25], vehicle-treated APPswePS1ΔE9 mice demonstrated a deterioration of object memory (Figure 3a), spatial memory (Figure 3b), and spatial working memory (Figure 3c) at the end of the 12-week treatment period, at the age of approximately 8.5 months. Our data showed that this cognitive deterioration of object memory, spatial memory, and spatial working memory in APPswePS1ΔE9 mice could be prevented by the supplementation of the lipid extract of *H. elongata* (*p* = 0.0260, *p* = 0.0147 and *p* = 0.0212, respectively) (Figure 3a–c). Upon supplementation with the *S. fusiforme* SCF extract, there was a trend toward prevention of the deterioration of object memory (*p* = 0.0570) (Figure 3a), spatial memory (*p* = 0.0855) (Figure 3b), and spatial working memory (*p* = 0.0522) (Figure 3c) in APPswePS1ΔE9 mice, but no statistical significance was reached.

### 3.4. Aβ Plaque Load in the Cortex and Hippocampus Remained Unaffected by Both Seaweed Extracts

**Figure 4:**
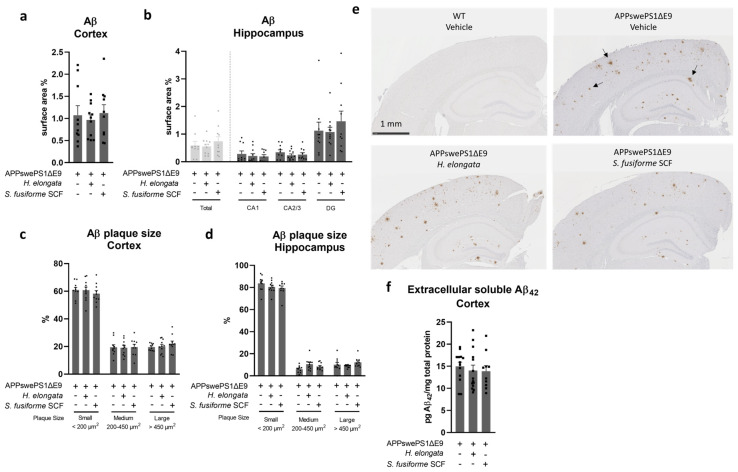
No effect of *H. elongata* lipid extract or *S. fusiforme* SCF extract on Aβ plaque load in APPswePS1ΔE9 mice. After staining brain slices for Aβ (*n* = 10–11 per group, 1 slide per animal), the surface area percentages of staining in the cortex (**a**), hippocampus, and hippocampal subfields CA1, CA2/3, and DG (**b**) were determined. The size distribution of the Aβ plaques (indicated with arrows in Figure (**e**)) was assessed by determining the percentages of small (<200 μm^2^), medium (200–450 μm^2^), and large (>450 μm^2^) plaques in the cortex (**c**) and hippocampus (**d**). Representative images of the Aβ staining are presented in Figure (**e**). The concentration of extracellular soluble Aβ_42_ in the cortex was determined with ELISA (**f**). Data are presented as mean ± SEM and were analyzed with one-way ANOVA (ordinary one-way ANOVA (with Dunnett’s multiple comparisons test) (**c**,**f**) or Kruskal–Wallis test (with Dunn’s multiple comparisons test) (**a**,**b**,**d**)). None of the differences between vehicle-treated and extract-treated mice were statistically significant.

No effect of the *H. elongata* lipid extract or *S. fusiforme* SCF extract was detected on the Aβ plaque load in the cortex (*p* = 0.8788), in the hippocampus (*p* = 0.8015), or in hippocampal subfields cornu ammonis 1 (CA1), cornu ammonis 2/3 (CA2/3), and dentate gyrus (DG) (*p* = 0.6613, *p* = 0.6270, and *p* = 0.8514, respectively) of APPswePS1ΔE9 mice (Figure 4a,b). Most of the plaques in the cortex and hippocampus were small-sized (<200 μm^2^), and this remained unaltered upon administration of *H. elongata* extract or *S. fusiforme* SCF extract (Figure 4c–e). The concentrations of extracellular soluble Aβ_42_ in the cortex also remained unaffected (F (2, 34) = 0.2560, *p* = 0.7756) (Figure 4f).

### 3.5. Microglia Marker Iba1 and Phagocytic Microglia/Macrophage Marker CD68 Decreased by H. elongata and S. fusiforme SCF Extracts

**Figure 5:**
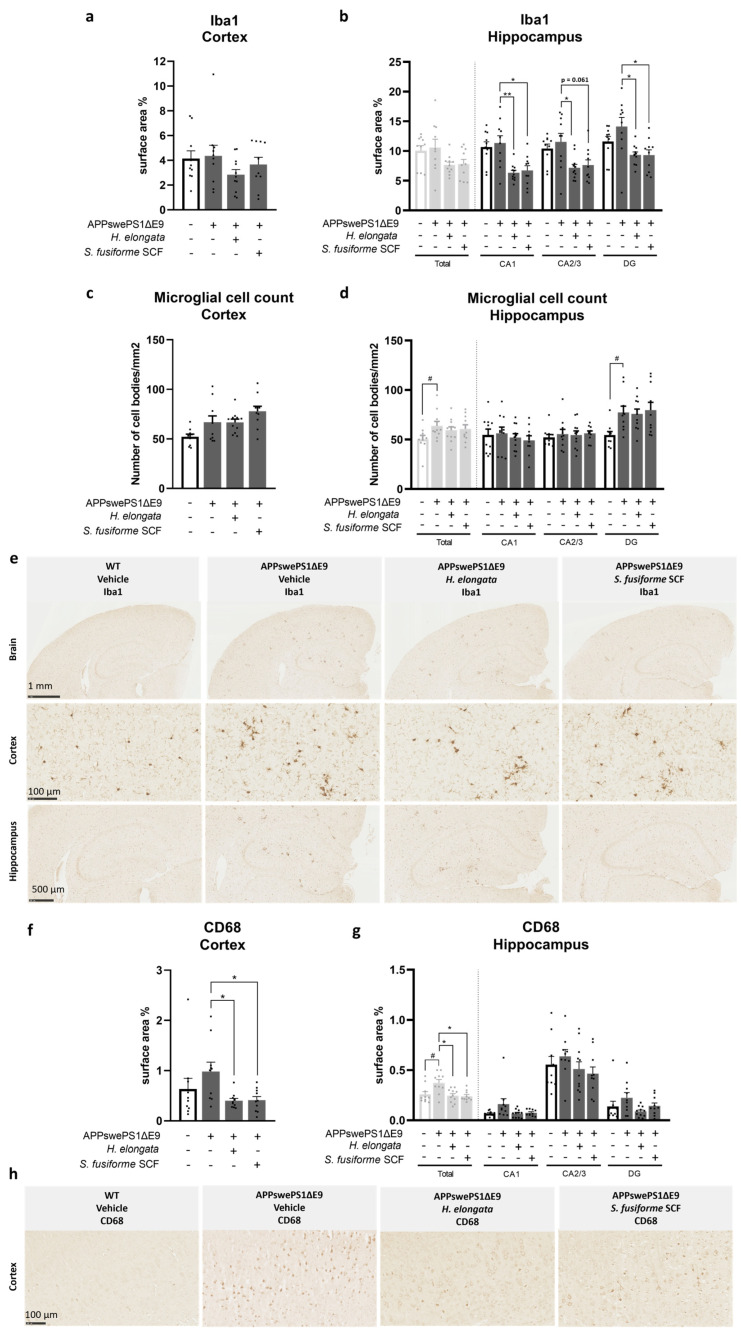
*H. elongata* lipid extract and *S. fusiforme* SCF extract decrease microglia marker Iba1 and phagocytic marker CD68 in the cortex and the hippocampus. After staining brain slices of WT and APPswePS1ΔE9 mice for Iba1 or CD68 (*n* = 10–11 per group, 3 cortical and 1 hippocampal image of 1 slide per animal), the relative cortical and hippocampal surface areas of Iba1 (**a**,**b**) and CD68 (**f**,**g**) staining were determined. The microglial cell count was determined by the number of cell bodies positively stained for Iba1 per mm^2^ (**c**,**d**). Representative images of the immunohistochemical staining of Iba1 and CD68 are presented in (**e**,**h**), respectively. Data are represented as mean ± SEM. Differences between vehicle-treated APPswePS1ΔE9 and WT mice were analyzed with a Mann–Whitney U test (# *p* < 0.05); Treatment effects in APPswePS1ΔE9 mice were analyzed with a Kruskal–Wallis test (with Dunn’s multiple comparisons test) (* *p* < 0.05, ** *p* < 0.01).

To examine treatment effects on inflammatory markers in the brain, we assessed the relative surface area (%) of the universal microglial marker Iba1 and the phagocytic microglia/macrophage marker CD68 in the cortex and hippocampus (Figure 5). Although Iba1 protein expression in the cortex (*p* = 0.7959) or hippocampus (*p* = 0.7959) of vehicle-treated APPswePS1ΔE9 and WT mice was not significantly different (Figure 5a,b), the microglial cell count, as determined by the number of cell bodies positively stained for Iba1, was higher in the total hippocampus (*p* = 0.0232) and hippocampal subfield DG (*p* = 0.0039) of APPswePS1ΔE9 mice than of WT mice (Figure 5d). The Iba1 positive-stained cells clustered in the cortex and hippocampus of APPswePS1ΔE9 mice, surrounding Aβ aggregates (Figure 5e). The CD68 protein expression was higher in the hippocampus of vehicle-treated APPswePS1ΔE9 mice compared to WT mice (*p* = 0.0355) (Figure 5g). This trend was also visible for the cortex but did not reach statistical significance (*p* = 0.0892) (Figure 5f).

Diet supplementation with *H. elongata* extract decreased Iba1 protein expression in the hippocampal subfields CA1, CA2/3, and DG of APPswePS1ΔE9 mice (*p* = 0.0048, *p* = 0.0414, and *p* = 0.0118, respectively) (Figure 5b). The *H. elongata* extract also decreased the induced CD68 expression observed in the cortex and hippocampus of vehicle-treated APPswePS1ΔE9 mice (*p* = 0.0119 and *p* = 0.0172, respectively) (Figure 5f,g).

Supplementation with *S. fusiforme* SCF extract also decreased Iba1 protein expression in hippocampal subfields CA1 and DG of APPswePS1ΔE9 mice (*p* = 0.0226 and *p* = 0.0183, respectively) (Figure 5b). Such a trend was also visible for the hippocampal subfield CA2/3 (*p* = 0.0609) (Figure 5b). The *S. fusiforme* SCF extract also appeared to decrease the induction of CD68 protein expression in the cortex and hippocampus of APPswePS1ΔE9 mice (*p* = 0.0226 and *p* = 0.0122, respectively) (Figure 5f,g).

### 3.6. H. elongata Extract and S. fusiforme SCF Extract Decreased the Astrocytic Marker GFAP in the Cortex of APPswePS1ΔE9 Mice but Increased GFAP in the Hippocampus

**Figure 6:**
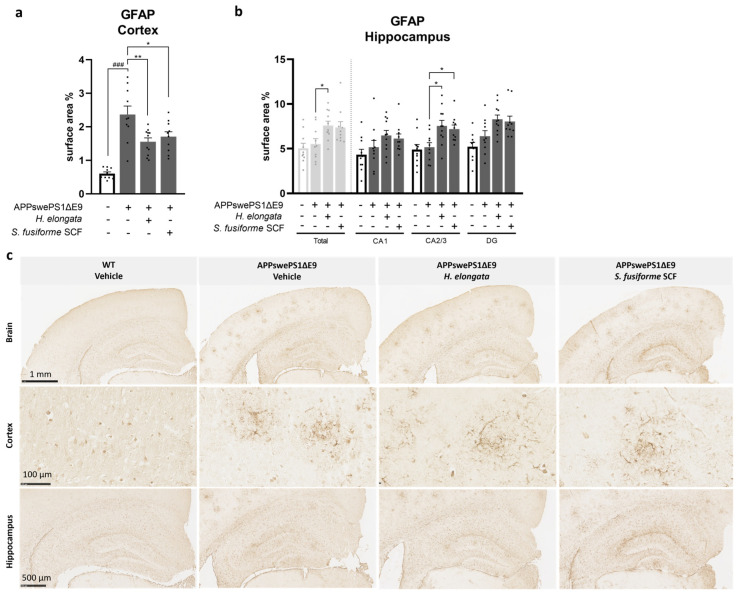
*H. elongata* extract and *S. fusiforme* SCF extract reduce the elevated astrocytic marker GFAP in the cortex of APPswePS1ΔE9 mice but increase GFAP in the hippocampus. The quantification of the immunohistochemical staining of GFAP in the cortex (**a**) and hippocampus (**b**) of WT and APPswePS1ΔE9 mice are presented as relative surface area. Representative images of the GFAP staining are presented in (**c**). Data are represented as mean ± SEM (*n* = 10–11 per group, 3 cortical and 1 hippocampal image of 1 slide per animal). Differences between vehicle-treated APPswePS1ΔE9 and WT mice were analyzed with an unpaired *t*-test (**a**) or Mann–Whitney U test (**b**) (### *p* < 0.001); Treatment effects in APPswePS1ΔE9 mice were analyzed with a one-way ANOVA (with Dunnett’s multiple comparisons test) (**a**) or a Kruskal–Wallis test (with Dunn’s multiple comparisons test) (**b**) (* *p* < 0.05, ** *p* < 0.01).

The effects of the seaweed extracts on the activation of astrocytes—also key players in the brain immune system—were assessed by determining the relative GFAP surface area (%) in the cortex and hippocampus (Figure 6). In vehicle-treated APPswePS1ΔE9 mice compared to WT mice, the cortical GFAP expression was elevated (*p* < 0.0001), while the hippocampal GFAP expression was similar (*p* = 0.5787) (Figure 6a,b). The GFAP-positive cells were clustered in the cortex and hippocampus of APPswePS1ΔE9 mice, as presented in Figure 6c.

Both *H. elongata* lipid and *S. fusiforme* SCF extracts reduced the increase in GFAP protein expression in the cortex of APPswePS1ΔE9 mice (*p* = 0.0054 and *p* = 0.0266, respectively) (Figure 6a). In contrast, *H. elongata* extract induced GFAP expression in the hippocampus of APPswePS1ΔE9 mice (*p* = 0.0220). A similar effect was observed for *H. elongata* in hippocampal subfields CA2/3 (*p* = 0.0106) and, although not statistically significant, in DG (*p* = 0.0868) (Figure 6b). While GFAP protein expression in the total hippocampus was not significantly affected by *S. fusiforme* SCF extract (*p* = 0.1101), GFAP expression in the hippocampal subfield CA2/3 was increased by supplementation with *S. fusiforme* SCF extract (*p* = 0.0308) (Figure 6b).

### 3.7. H. elongata Extract Reduced the Production of Inflammatory Cytokines in THP-1-Derived Macrophages While the S. fusiforme SCF Extract Only Decreased TNFα

**Figure 7:**
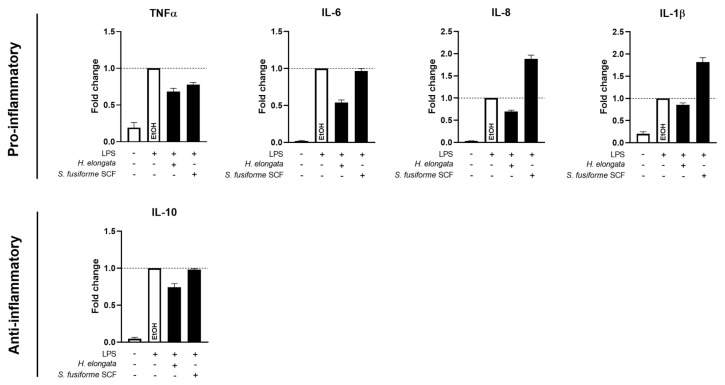
Pro- and anti-inflammatory cytokine production by THP-1-derived macrophages. The concentrations of pro-inflammatory cytokines TNFα, IL-6, IL-8, and IL-1β and anti-inflammatory cytokine IL-10 were determined in the medium of LPS-stimulated THP-1-derived macrophages after incubation with *H. elongata* and *S. fusiforme* SCF extracts for 24 h. The extracts were added based on their saringosterol concentrations with a final saringosterol concentration of 0.6 µM. Data from three independent experiments are expressed as fold change relative to the EtOH-incubated cells and presented as mean ± SEM (*n* = 3–5).

To further evaluate the immunomodulatory effects of the extracts, we examined their effects on the cytokine production in LPS-stimulated THP-1-derived macrophages. The *H. elongata* extract decreased the production of pro-inflammatory cytokines TNFα, IL-6, and IL-8 in the medium of LPS-stimulated THP-1-derived macrophages, which further supports the anti-inflammatory properties of the *H. elongata* lipid extract. The production of anti-inflammatory cytokine IL-10 was slightly reduced upon incubation with the *H. elongata* extract (Figure 7). The *S. fusiforme* SCF extract slightly reduced the TNFα concentrations. However, the production of IL-6 and IL-10 remained unaffected, and IL-8 and IL-1β were upregulated (Figure 7).

### 3.8. Promotion of Cholesterol Efflux in THP-1-Derived Macrophages

**Figure 8:**
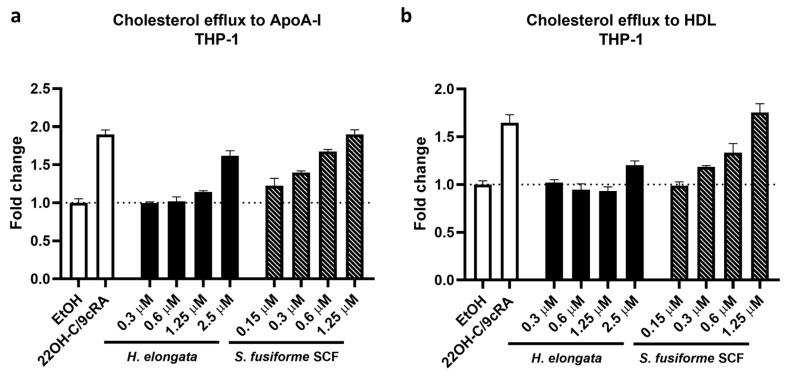
Effect of *H. elongata* and *S. fusiforme* SCF extracts on cholesterol efflux in THP-1-derived macrophages. Cholesterol efflux from THP-1-derived macrophages to ApoA-I (10 µg/mL) (**a**) and HDL (12.5 µg/mL) (**b**) was determined after 20 h incubation with *H. elongata* lipid extract or *S. fusiforme* SCF extract. The extracts were added based on their saringosterol concentration (on the *X*-axis). Cells incubated with 22OH-C/9cRA (5 µg/mL/10 µM) served as a positive control. The data are expressed as cholesterol efflux relative to the efflux by EtOH-incubated cells. Bars represent mean ± SEM (*n* = 3).

Because the cellular cholesterol content may affect the cytokine production of macrophages, we determined the effect of the extracts of *H. elongata* and *S. fusiforme* on cholesterol efflux in THP-1-derived macrophages [15]. In line with our previous findings that *H. elongata* and *S. fusiforme* extracts upregulate the expression of genes involved in cholesterol efflux [28], the *H. elongata* lipid extract and *S. fusiforme* SCF extract promoted the efflux of cholesterol from THP-1-derived macrophages. The *H. elongata* extract promoted the cholesterol efflux to ApoA-I and, to a lesser extent, to HDL, but only at a saringosterol concentration of 2.5 µM (Figure 8a,b). The *S. fusiforme* SCF extract promoted the efflux of cholesterol from THP-1-derived macrophages to ApoA-I and HDL in a dose-dependent manner (Figure 8a,b).

### 3.9. H. elongata May Promote Early Oligodendrocyte Maturation

**Figure 9:**
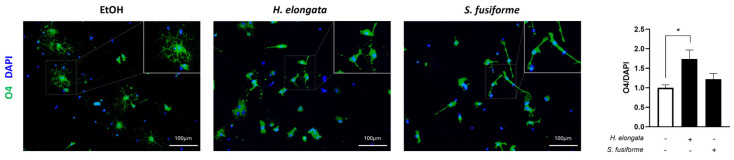
Expression of oligodendrocyte marker O4 by OPCs incubated with *H. elongata* and *S. fusiforme* lipid extracts. The surface area percentage of O4 staining, as a marker of pre-oligodendrocytes, was determined on cultured OPCs incubated with lipid extracts of *H. elongata* and *S. fusiforme*. The extracts were added based on saringosterol concentration, with a final saringosterol concentration of 0.6 µM. The O4 expression was normalized to the surface area percentage of a DAPI staining and expressed as fold change relative to ethanol-incubated cells. Representative images of the O4 and DAPI staining are presented. Data are presented as mean ± SEM (*n* = 5) and were analyzed with one-way ANOVA (with Dunnett’s multiple comparisons test) (* *p* < 0.05).

By stimulating cholesterol efflux [42,43] and inhibiting inflammatory responses [43,44,45], the *H. elongata* and *S. fusiforme* extracts may favor oligodendrocyte differentiation and, thereby, potentially enhance (re-)myelination [46,47]. Such interventions targeting myelin volume may enhance cognitive reserves and prevent AD-related symptoms [42,48]. We determined the effects of *H. elongata* and *S. fusiforme* lipid extracts on the expression of the early oligodendrocyte marker O4 in OPCs (Figure 9). The *H. elongata* lipid extract increased the O4 expression (*p* = 0.0143), suggesting a positive effect on early OPC differentiation, while the *S. fusiforme* extract showed no effect (*p* = 0.5539).

### 3.10. Effects of 1-Week Administration of H. elongata Extract on Hippocampal Transcriptome

**Figure 10:**
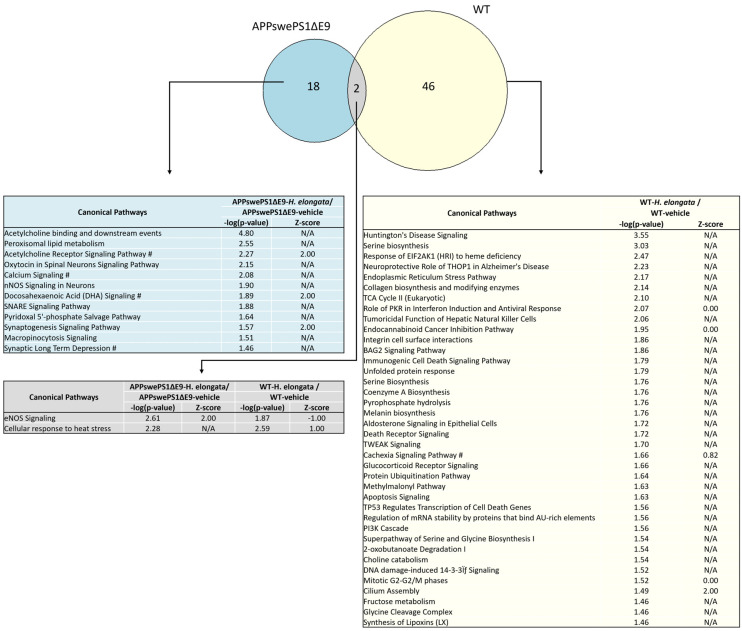
Pathways in the hippocampus of APPswePS1ΔE9 and WT mice affected by supplementation with *H. elongata* lipid extract. Venn diagram displaying the overlap of pathways that were affected by *H. elongata* lipid extract in APPswePS1ΔE9 and WT mice. #: Pathways differentially regulated in WT and APPswePS1ΔE9 mice.

We conducted a transcriptome analysis on the hippocampus of both APPswePS1ΔE9 and WT mice after 1 week of diet supplementation with *H. elongata* extract to investigate acute effects on pathways that may have played a role in the beneficial effects of *H. elongata* lipid extract on cognitive decline in the APPswePS1ΔE9 mouse model of AD (Figure 10 and Table S1). Diet supplementation with the *H. elongata* extract exhibited distinct impacts in both APPswePS1ΔE9 and WT mice (Figure 10 and Table S1). In APPswePS1ΔE9 mice, *H. elongata* extract affected pathways related to neurotransmission, including acetylcholine and synaptogenesis signaling, and synaptic long-term depression (Figure 10 and Table S1). Four of the canonical pathways exclusively affected in the APPswePS1ΔE9 mice by *H. elongata* extract were calcium signaling, acetylcholine receptor signaling pathway, synaptic long-term depression, and docosahexaenoic acid (DHA) signaling. Notably, in WT mice, the *H. elongata* extract had a dissimilar effect suggesting that the beneficial effects of *H. elongata* on these pathways are specific in the context of the disease. In WT mice, the *H. elongata* extract affected pathways related to Huntington’s Disease and endoplasmatic reticulum (ER) stress (Figure 10 and Table S1).

### 3.11. Phytosterol Concentrations after Diet Supplementation with H. elongata Lipid Extract and S. fusiforme SCF Extract

Next, we determined the effect of 12 weeks of diet supplementation with *H. elongata* lipid extract and *S. fusiforme* SCF extract on (phyto)sterols, in the cerebellum, liver, and serum of APPswePS1ΔE9 mice to confirm the presence of saringosterol in the brain and to assess the effects of the treatments on sterol homeostasis, which is known to be affected in AD.

**Figure 11:**
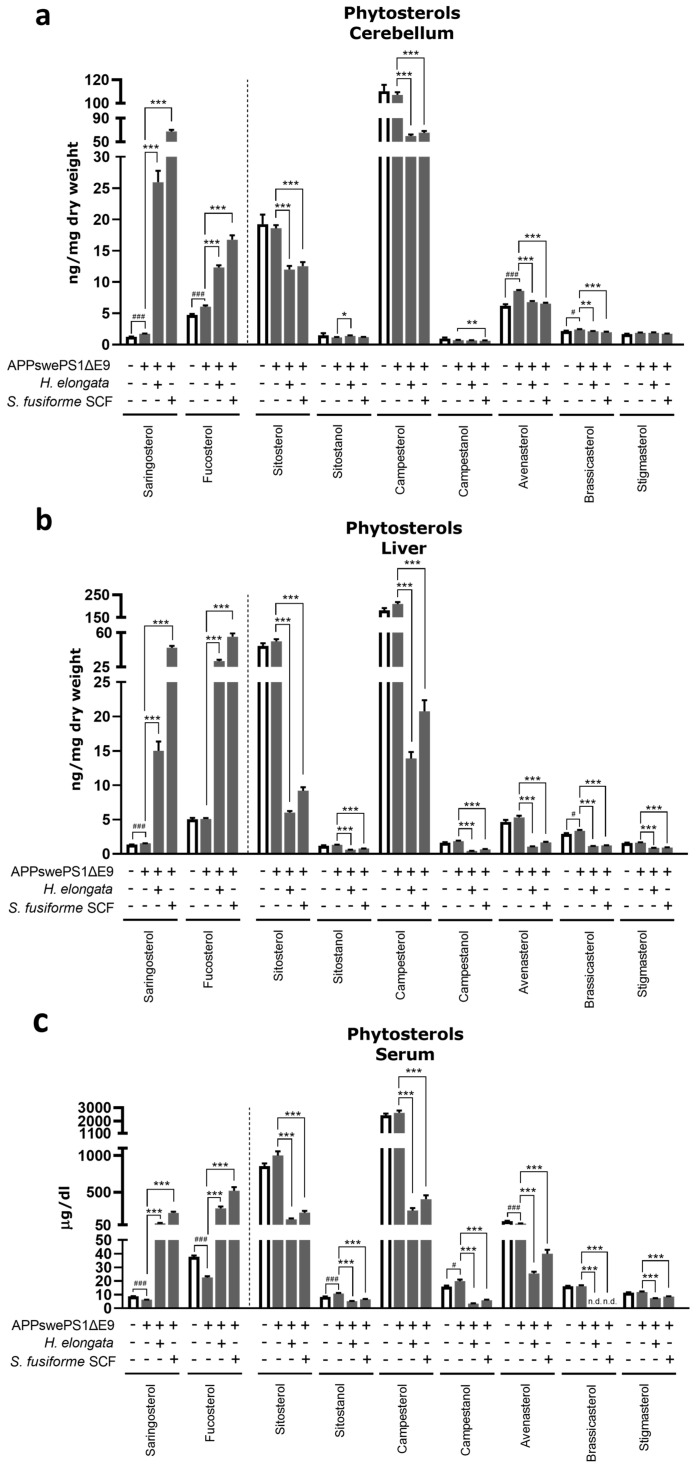
Phytosterol concentrations in the cerebellum (**a**), liver (**b**), and serum (**c**) of WT and APPswePS1ΔE9 mice upon diet supplementation with *H. elongata* and *S. fusiforme* SCF extract. Data are presented as mean ± SEM (*n* = 10–13 per group). Differences between vehicle-treated APPswePS1ΔE9 and WT mice were analyzed with an unpaired *t*-test (# *p* < 0.05, ### *p* < 0.001); Treatment effects in APPswePS1ΔE9 mice were analyzed with a one-way ANOVA (with Dunnett’s multiple comparisons test) (* *p* < 0.05, ** *p* < 0.01, *** *p* < 0.001). n.d.: not detectable.

Diet supplementation with either *H. elongata* lipid or *S. fusiforme* SCF extract increased the absolute concentrations of saringosterol and fucosterol as well as their concentrations relative to cholesterol in the cerebellum, liver, and serum (Figure 11a–c and Table S2). In contrast, the concentrations of the majority of the other detected phytosterols were decreased (Figure 11a–c). The ratios of campesterol, campestanol, sitosterol, sitostanol, avenasterol, brassicasterol, and stigmasterol to cholesterol in the liver and serum were reduced upon supplementation with the extracts (Table S2).

### 3.12. Cholesterol and Cholesterol Precursors and Metabolites

**Figure 12:**
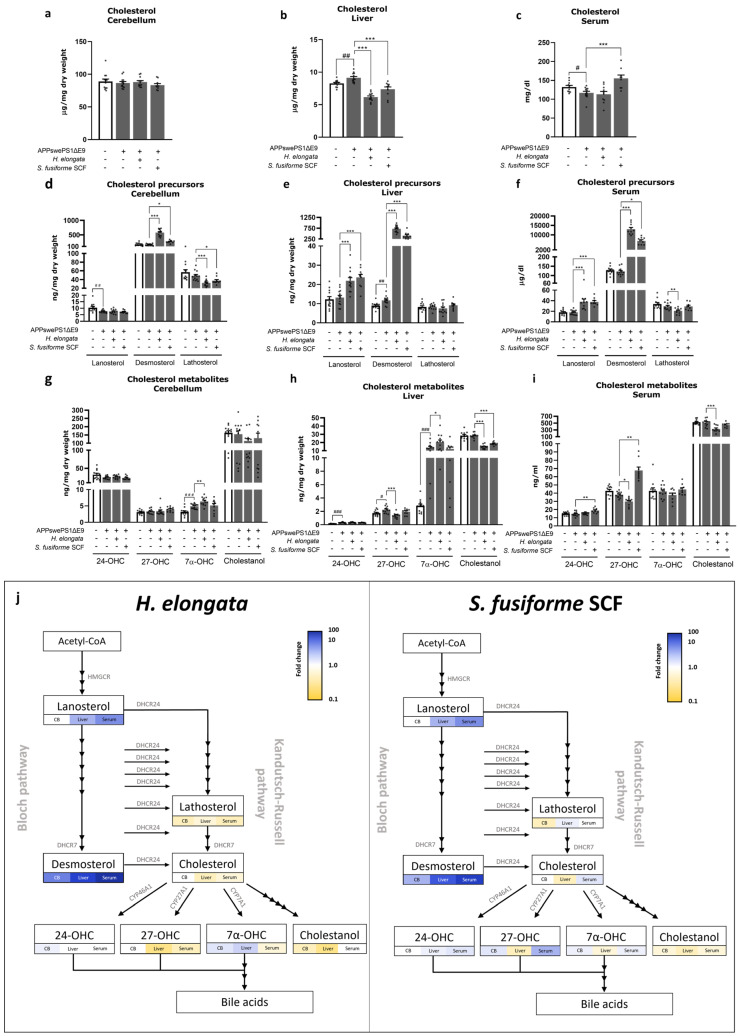
The concentrations of cholesterol (**a**–**c**), cholesterol precursors (**d**–**f**), and cholesterol metabolites (**g**–**i**) in the cerebellum, liver, and serum of WT and APPswePS1ΔE9 mice upon supplementation with *H. elongata* extract and *S. fusiforme* SCF extract. (**j**) presents the fold changes of extract-treated mice relative to vehicle-treated mice in the cholesterol synthesis and oxidation pathways. Data are represented as mean ± SEM (*n* = 9–13 per group). Differences between vehicle-treated APPswePS1ΔE9 and WT mice were analyzed with an unpaired *t*-test (**a**–**c**,**e**,**h**) or a Mann–Whitney U test (**d**,**f**,**g**,**i**) (# *p* < 0.05, ## *p* < 0.01, ### *p* < 0.001); Treatment effects in APPswePS1ΔE9 mice were analyzed with a one-way ANOVA (**a**–**c**,**e**,**h**) or a Kruskal–Wallis test (**d**,**f**,**g**,**i**) (with Dunnett’s/Dunn’s multiple comparisons test) (* *p* < 0.05, ** *p* < 0.01, *** *p* < 0.001).

The differences in concentrations of cholesterol, its precursors (lanosterol, desmosterol and lathosterol), and metabolites (24-hydroxycholesterol (24-OHC), 27-hydroxycholesterol (27-OHC), 7α-hydroxycholesterol (7α-OHC), and cholestanol) between vehicle-treated APPswePS1ΔE9 and WT mice are shown in Figure 12 and Table S2.

Supplementation with *H. elongata* lipid extract and *S. fusiforme* SCF extract decreased the cholesterol concentration in the liver of APPswePS1ΔE9 mice (both *p* < 0.0001) (Figure 12b), and the *S. fusiforme* SCF extract increased the cholesterol concentration in serum (*p* = 0.0005) (Figure 12c), while there was no effect in the cerebellum (Figure 12a).

Supplementation with both *H. elongata* extract and *S. fusiforme* SCF extract notably increased the concentration of desmosterol, the direct precursor of cholesterol, in the cerebellum (*p* < 0.0001 and *p* = 0.0054, respectively), liver (both *p* < 0.0001), and serum (*p* < 0.0001 and *p* = 0.0126, respectively) of APPswePS1ΔE9 mice (Figure 12d–f). Accordingly, the *H. elongata* and *S. fusiforme* SCF extracts remarkably increased the desmosterol/cholesterol ratios up to, respectively, 120-fold and 40-fold increases in the serum (*p* < 0.0001) (Table S2). Additionally, the ratio of lanosterol/cholesterol was increased in the liver (both *p* < 0.0001) and serum (*p* < 0.0001 and *p* = 0.0108, respectively) by *H. elongata* and *S. fusiforme* SCF extract (Table S2). However, the *H. elongata* extract and *S. fusiforme* SCF extract decreased the ratio of lathosterol/cholesterol in the cerebellum (*p* < 0.0001 and *p* = 0.0337, respectively) and serum (*p* = 0.0014 and *p* = 0.0033, respectively) but increased it in the liver (*p* = 0.0213 and *p* = 0.0313) (Table S2).

The ratio of 24-OHC/cholesterol in the liver was increased by *H. elongata* (*p* < 0.0001) but not by *S. fusiforme* SCF (*p* = 0.1652) (Table S2). The ratio of 27-OHC/cholesterol in the serum was increased by *S. fusiforme* SCF (*p* = 0.0118) (Table S2). Although the *H. elongata* extract increased 7α-OHC/cholesterol in the cerebellum and especially in the liver (*p* = 0.0298 and *p* = 0.0029, respectively), the *S. fusiforme* SCF extract decreased this ratio in the serum (*p* = 0.0094) (Table S2). The cholestanol/cholesterol ratio was decreased by the *H. elongata* and *S. fusiforme* SCF extracts in both the liver (*p* = 0.0016 and *p* = 0.0179, respectively) and serum (both *p* < 0.0001) (Table S2).

A schematic overview of the effects of the extracts on the concentrations of cholesterol and its precursors and metabolites in the cholesterol synthesis and oxidation pathways is presented in Figure 12j. The ratios of these sterols to cholesterol relative to vehicle-treated WT mice are presented in Table S2.

The effects of 12 weeks of diet supplementation with *H. elongata* extract on the sterol profiles in the cerebellum, liver, and serum (Figure 12) resembled the patterns observed after 1 week of supplementation with *H. elongata* extract (Figure S3). However, the alterations in sterol levels were less pronounced compared to the extended 12-week supplementation. This is in accordance with the relatively low concentrations of saringosterol (3.2 ± 0.6 ng/mg hippocampus, 6.2 ± 1.1 ng/mg cerebellum, 4.9 ± 0.8 ng/mg liver, and 25.7 ± 9.1 µg/dL serum) and fucosterol (3.0 ± 0.6 hippocampus, 3.9 ± 0.6 ng/mg cerebellum, 21.7 ± 3.9 ng/mg liver, and 227.1 ± 57.3 µg/dL serum) after 1 week of supplementation compared to 12 weeks of supplementation (saringosterol: 26.0 ± 6.3 ng/mg cerebellum, 15.0 ± 4.6 ng/mg liver, and 71.6 ± 22.2 µg/dL serum; fucosterol: 12.3 ± 1.2 ng/mg cerebellum, 31.1 ± 4.7 ng/mg liver, and 277.6 ± 78.2 µg/dL serum).

### 3.13. The Potential of H. elongata Lipid Extract and S. fusiforme SCF Extract to Suppress Hepatic Inflammation

The elevated hepatic neutral lipid levels in the liver of APPswePS1ΔE9 mice (Figure S1c) may trigger an inflammatory response, whereas the *H. elongata* extract and *S. fusiforme* SCF extract potentially suppress hepatic inflammation, possibly by elevating desmosterol levels in the liver. We determined the relative surface area (%) of the macrophage marker F4/80 in the liver of WT and APPswePS1ΔE9 mice after 12 weeks of supplementation with the *H. elongata* lipid extract and *S. fusiforme* SCF extract (Figure S4). The protein expression of the macrophage marker F4/80 in the liver of APPswePS1ΔE9 mice was not elevated compared to WT mice (*p* = 0.8344) and remained unaffected by *H. elongata* and *S. fusiforme* SCF extracts (*p* = 0.8344) (Figure S4).

### 3.14. H. elongata and S. fusiforme SCF Extracts Reduce AD-Related Tau Pathology In Vitro

Cholesterol metabolism has also been linked to the precipitation of tau and, consequently, to tau pathology [49,50], and LXR activation ameliorated tau pathology in a mouse model of tauopathy [51]. Therefore, we evaluated the effect of *H. elongata* lipid extract, *S. fusiforme* SCF extract, seaweed-derived oxyphytosterol saringosterol, and desmosterol on AD-related tau pathology in differentiated SH-SY5Y cells (Figure S5). The AD-associated variant of tau phosphorylated at Ser202 and Thr205 showed a dose-dependent decrease upon incubation with the *H. elongata* lipid extract, up to a 62% reduction at its highest dose (with a saringosterol concentration of 4.8 µM) (Figure S5a). In contrast, saringosterol incubation increased the concentrations of this *p*-tau variant in a dose-dependent manner (Figure S5a). The *S. fusiforme* SCF extract and LXR agonist T0901317 did not affect these p-tau concentrations (Figure S5a). Desmosterol also did not affect the p-tau concentration. The total tau concentration was decreased by the *H. elongata* extract but was not affected by *S. fusiforme* SCF extract, saringosterol, or desmosterol (Figure S5b). The elevated p-tau in the forebrain of APPswePS1ΔE9 mice as compared to WT mice (*p* = 0.0461) was not affected by diet supplementation with *H. elongata* lipid extract for 12 weeks (*p* = 0.3217) (Figure S5c).

## 4. Discussion

We investigated the effects of a lipid extract of *H. elongata* and an SCF extract of *S. fusiforme* on cognitive performance and the development of AD-related pathology in APPswePS1ΔE9 mice. Our data demonstrate that supplementation of the diet with extracts of *H. elongata* or *S. fusiforme* has the potential to prevent or postpone cognitive decline and to reduce the Aβ-induced activation of (phagocytic) microglia and astrocytes in cerebral tissue of APPswePS1ΔE9 mice. The extracts affected sterol homeostasis in the brain and liver, as indicated by alterations in the concentrations of cholesterol and its precursors and metabolites, which may have contributed to their neuroprotective effects. Unlike synthetic LXR agonists, the seaweed extracts did not significantly induce hepatic steatosis or hypertriglyceridemia. The notable decrease in weight gain among mice receiving the *H. elongata* extract highlights the potential of *H. elongata* as a therapeutic intervention for metabolic conditions linked to increased body weight.

### 4.1. Prevention of Cognitive Decline by H. elongata Extract, and Possibly by S. fusiforme SCF Extract, with No Impact on Aβ plaque Load

Our data demonstrate the prevention of cognitive decline with the lipid extract of *H. elongata*. A trend toward this beneficial effect was also observed for the SCF extract of *S. fusiforme*. Statistical significance with the *S. fusiforme* SCF extract may not have been reached because of the relatively small sample size of *S. fusiforme* SCF extract-treated APPswePS1ΔE9 mice and the relatively short duration of the experiments (12 weeks). The amelioration of cognitive decline without affecting soluble or insoluble Aβ is consistent with previous results observed with the oxyphytosterol 24(*S*)-saringosterol [24] and the synthetic LXR agonist T0901317 [19] but is in contrast with the decrease in insoluble Aβ reported in APPswePS1ΔE9 mice supplemented with crude *S. fusiforme* or its lipid extract [25]. However, many elderly people accumulate Aβ plaques in the brain without suffering from any AD symptoms [52]. In addition, several studies reported preservation of cognitive decline despite the presence of Aβ plaques [19,24,53], suggesting that insoluble Aβ plaques may not directly cause clinical symptoms and that decreasing these Aβ plaques is not crucial for ameliorating AD symptoms.

### 4.2. Effects of H. elongata and S. fusiforme Extracts on Cholesterol Metabolism: Cholesterol-Lowering Effects and a Notable Increase in Endogenous LXR Agonist Desmosterol Possibly through DHCR24 Inhibition

Upon supplementation with the extracts, the concentrations of the seaweed-derived phytosterols saringosterol and fucosterol in the serum, cerebellum, and liver increased, while the concentrations of other phytosterols commonly present in chow as well as the hepatic cholesterol concentration decreased. This decrease in common phytosterols and cholesterol may be due to competition with the relatively high amounts of saringosterol and fucosterol for incorporation into mixed micelles in the intestine leading to diminished absorption. Another possible contributing factor is the enhancement of sterol excretion into the bile through the upregulation of ATP-binding cassette (ABC) transporters ABCG5 and ABCG8 upon LXR activation [54,55]. Additionally, the concentration of cholesterol in the liver of *H. elongata*- and *S. fusiforme*-treated mice may be decreased as a result of an inhibitory effect of (oxy)sterols on sterol regulatory element-binding protein (SREBP) activation [56], as suggested by the decrease in *SREBF1* expression. In addition, cholesterol may be reduced due to the upregulation of desmosterol, which could inhibit SREBP2 processing and reduce the expression of its target gene *3-hydroxy-3-methylglutaryl-CoA reductase* (*HMGCR*), thereby reducing cholesterol synthesis [57]. In addition, supplementation with *H. elongata* extract increased the concentration of the cholesterol metabolite and bile acid precursor 7α-OHC, which may result from LXRα-induced upregulation of CYP7A1 expression [58,59] facilitating the conversion of cholesterol to 7α-OHC, generally considered the rate-limiting step in bile acid synthesis. The increased conversion of cholesterol into 7α-OHC may also have resulted in the decline in hepatic cholesterol, cholestanol, and 27-OHC observed with the *H. elongata* extract. The 27-OHC concentration may also be reduced as a result of enhanced bile acid synthesis through the alternative pathway implicating the involvement of CYP7B1. However, this is unlikely as LXR activation has previously been demonstrated to suppress the expression of CYP7B1 and is, therefore, expected to rather increase the 27-OHC concentrations [60], as observed in serum upon diet supplementation with the *S. fusiforme* SCF extract. Given that hypercholesterolemia elevates the susceptibility to cardiovascular and neurodegenerative conditions [61], the reduction in cholesterol facilitated by the extracts may offer additional advantages.

Diet supplementation with either the *H. elongata* or *S. fusiforme* SCF extract increased the concentrations of lanosterol and, especially, of desmosterol in the liver, serum, and cerebellum, while concentrations of lathosterol decreased or remained unchanged. Cholesterol can be synthesized via two pathways, the Bloch and Kandutsch–Russell pathways, that are connected by the enzyme 24-dehydrocholesterol reductase (DHCR24) [62]. Lanosterol is the first sterol intermediate in cholesterol synthesis that can be converted into cholesterol via the Bloch and Kandutsch–Russell pathways; desmosterol is the immediate precursor to cholesterol in the Bloch pathway, and lathosterol is an intermediate product of cholesterol synthesis in the Kandutsch–Russell pathway. Our data demonstrating elevated lanosterol and desmosterol concentrations and decreased or unaffected lathosterol concentrations are, therefore, suggestive of an inhibitory effect of the extracts on DHCR24. This is in line with the decrease in *DHCR24* expression demonstrated with lipid extracts of *H. elongata* and *S. fusiforme* on the transcriptional level in an astrocytoma cell line [28]. DHCR24 inhibition would result in a reduced flux through the Kandutsch–Russell pathway and reduced conversion of desmosterol into cholesterol. The downregulation of DHCR24 might therefore also have contributed to the desmosterol accumulation as well as the decreased cholesterol concentration observed in the liver. DHCR24 plays a crucial role in maintaining cholesterol homeostasis by controlling cholesterol synthesis and can be inhibited by certain phytosterols [63], side-chain oxysterols [64], as well as the synthetic LXR agonist N,N-dimethyl-3β-hydroxycholenamide (DMHCA) [65]. DHCR24 transcription is suggested to be under the control of SREBPs [66,67], hence the suggested DHCR24 inhibition might result from the inhibition of SREBP. The increase in cholesterol concentration in the serum of *S. fusiforme* SCF-treated APPswePS1ΔE9 mice could be the result of an increased ABCA1-mediated efflux of cholesterol to HDL or an increased efflux of cholesterol from the liver to VLDL, which might also have decreased the hepatic cholesterol concentration.

### 4.3. Human Mutant APPswe and PSENΔE9 Genes Affect Besides the Brain Also the Peripheral System

Interestingly, the concentration of 7α-OHC was significantly higher in the liver of APPswePS1ΔE9 than in WT mice. The APPswe and PSEN1ΔE9 genes in the APPswePS1ΔE9 mouse model are under the control of the mouse prion promoter, directing the expression of these genes primarily to neurons. However, we demonstrated that PSENΔE9 is also expressed in the liver of APPswePS1ΔE9 mice (Figure S6), likely because the prion promotor is also expressed in the liver, e.g., due to the presence of neurons in the liver [68]. In this way, the human mutant APPswe and PSENΔE9 genes affect not only the brain but also the peripheral system, as was previously demonstrated for both the liver and bladder [69,70]. The expression of these genes in the liver may influence the hepatic metabolism, thereby affecting, e.g., the bile acid precursor 7α-OHC. In line with our finding, alterations in bile acid synthesis have previously been reported for two other APP-related AD models with one model displaying increased 7α-OHC concentrations in the liver [71]. Moreover, Nho et al. [72] demonstrated associations between altered bile acid profiles and AD pathologies such as Aβ, p-tau, and cerebral atrophy. Together, these data suggest an association between AD-related pathophysiology and bile acid metabolism. The associations of APP with metabolic diseases, including type 2 diabetes, obesity, non-alcoholic fatty liver disease, and cardiovascular disease [69,73], further support the extracerebral effects of APP.

### 4.4. Anti-Inflammatory Effects of the Seaweed Extracts on Glial Cells and THP-1 Derived Macrophages: Potential Contribution of Promoted Cholesterol Efflux and Upregulated Desmosterol Concentrations

AD is strongly associated with persistent neuroinflammation caused by the excessive activation of microglia and astrocytes [13,74]. Since LXR activation, as well as seaweed-derived oxyphytosterol 24(*S*)-saringosterol, exerts anti-inflammatory effects [12,13,24], we investigated the impact of *H. elongata* lipid extract and *S. fusiforme* SCF extract on microglial and astrocytic markers. The observed reduction of microglial markers Iba1 and CD68 in the brain tissue of APPswePS1ΔE9 mice upon diet supplementation with either *H. elongata* extract or *S. fusiforme* SCF extract suggests a reduction in microglial activity, signifying a reduction in AD-related neuroinflammation. This is further supported by the reduced production of pro-inflammatory cytokines (TNFα, IL-6, and IL-8) by LPS-stimulated THP-1-derived macrophages in response to the *H. elongata* extract and reduced TNFα production in response to the *S. fusiforme* SCF extract. These findings are in line with the literature proposing anti-inflammatory effects of *S. fusiforme* and other seaweed species [75]. The observed reduction in protein expression of Iba1, which is considered a general marker of microglia and whose expression is thought to increase with microglial activation, suggests an overall reduction in microglial activity. The extract-induced decrease in the expression of CD68—a lysosomal marker and therefore considered a marker of activated phagocytic microglia and macrophages—suggests a reduced phagocytic activity of these inflammation-associated cells. Additionally, this reduction in CD68 may reflect a reduction in phagocyte infiltration [76]. By reducing microglial and macrophage activity and decreasing the production of pro-inflammatory cytokines, the extracts may be able to reduce inflammation-induced neuronal damage.

The elevated expression of the astrocytic marker GFAP in the cortex of APPswePS1ΔE9 mice, compared to WT mice, is suggestive of abnormal reactive astrogliosis, which is observed in neurodegenerative and non-neurodegenerative neurological conditions [77,78,79,80,81,82]. The elevated cortical GFAP expression was decreased upon diet supplementation with *H. elongata* and *S. fusiforme*, suggesting a reduction in cortical reactive astrocytes. On the contrary, the extracts increased the GFAP expression in the hippocampus of APPswePS1ΔE9 mice, which may suggest enhanced astrocyte activation or reactivity. The role of astrocyte activity in neurodegeneration is multifaceted, encompassing both advantageous and adverse effects. Astrocyte activation may exacerbate inflammatory activity; however, it may also suppress inflammation and enhance neuronal health by augmenting the neuroprotective functions of astrocytes, thereby contributing to tissue repair [83,84]. Considering the concurrent decrease in Iba1 expression, it can be speculated that the elevated GFAP levels have a protective rather than detrimental effect. It is essential to note that astrocytic marker GFAP alone might be insufficient to categorize astrocytes as reactive, given its broad applicability as a general astrocytic marker. Assessment of the presence of distinct astrocyte subtypes may contribute to a more nuanced understanding.

Our results of diet supplementation with *H. elongata* and *S. fusiforme* SCF extracts on microglial and astrocytic markers imply the potential anti-inflammatory effects of the extracts. Possibly, the ability of the extracts to promote cholesterol efflux—thereby facilitating neuronal cholesterol supply and reducing the cytotoxic accumulation of intracellular lipids [15]—contributed to these effects. Alternatively, the significant increase in desmosterol contributed to the reduction in these inflammation-related markers. Desmosterol is an endogenous LXR agonist that is known to facilitate the resolution of pro-inflammatory phenotypes through the suppression of inflammatory response genes by its effects on LXR [46,85,86]. The synthetic DHCR24 inhibitor SH42 has previously been reported to increase desmosterol levels causing an LXRα-dependent decrease in liver inflammation by preventing Kupffer cell activation and monocyte infiltration in a mouse model of human-like non-alcoholic fatty liver disease (NAFLD) and non-alcoholic steatohepatitis (NASH) [87]. Therefore, desmosterol may be involved in mediating the effects of the seaweed extracts on the glial markers. In addition, these data highlight the potential application of the *H. elongata* and *S. fusiforme* extracts in the context of NAFLD and NASH. Although we did not detect an effect of the *H. elongata* and *S. fusiforme* SCF extracts on the expression of the macrophage marker F4/80 in the liver of APPswePS1ΔE9 mice, it is worthwhile investigating the effects of these extracts on inflammation markers in models that do show liver inflammation.

The transcriptional analysis conducted on the hippocampus of APPswePS1ΔE9 mice indicated a potential influence of the *H. elongata* lipid extract on acetylcholine signaling. In accordance with the findings in AD patients [88], the acetylcholine signaling pathway in AD mouse model APPswePS1ΔE9 was altered. Acetylcholine signaling could promote neurotransmission and synaptic plasticity but is also known to decrease the inflammatory phenotype and the activation of macrophages and glial cells [88,89]. Through the promotion of acetylcholine signaling, the *H. elongata* extract may have affected the inflammation-related gliosis observed in the brain of APPswePS1ΔE9 mice.

### 4.5. H. elongata Extract Modulated Weight Gain in APPswePS1ΔE9 Mice: Implications for Metabolic and Neurodegenerative Conditions

Consistent with previous studies [90], the APPswePS1ΔE9 mice gained more weight throughout the experiment than the WT mice. The diminished weight gain of *H. elongata*-supplemented APPswePS1ΔE9 mice throughout the experiment emphasizes the potential of *H. elongata* as a therapeutic approach for metabolic diseases related to elevated body weight, hypertriglyceridemia, and hepatic steatosis. This decrease in weight gain may have contributed to the observed effects of *H. elongata* extract on neuro-inflammatory markers, since caloric restriction has demonstrated neuroprotective effects in APP/PS1 transgenic mice, e.g., improved neuronal activity and cognitive performance and reduced Aβ load, microgliosis, and astrocyte activation [91,92,93]. However, the *S. fusiforme* SCF extract exerted comparable effects on microglia and astrocyte markers without affecting weight gain, thereby likely excluding an effect of the latter.

### 4.6. No Adverse Effects of Seaweed Extracts on Triglyceride Content in Liver or Serum

In contrast with most tested synthetic LXR agonists [21,22,23], adverse effects on the hepatic lipid status were not detected upon diet supplementation with *H. elongata* and *S. fusiforme* SCF extracts, despite their ability to activate both LXRα and LXRβ. This is possibly the result of the inhibitory effects of seaweed-derived oxysterols or desmosterol on SREBP activation [56,57], as suggested by the decrease in expression of *SREBF1*. The decrease in relative VLDL levels in the serum observed with the *S. fusiforme* SCF extract may be attributed to a reduced (SREBP-controlled) triglyceride synthesis in the liver. Alternatively, the *S. fusiforme* SCF extract may promote lipolysis—e.g., by upregulation of lipoprotein lipase (LPL) expression as demonstrated upon LXR activation [94]—thereby decreasing VLDL and increasing LDL concentrations.

### 4.7. A Potential Role for H. elongata in Promoting Myelination

Reduced myelin volume and integrity—observed in infants carrying *APOE4*—are believed to impact the onset of AD by increasing the susceptibility to Aβ, tau, and neuroinflammatory toxicity [42,48]. Therefore, interventions enhancing myelin integrity may benefit cognitive deterioration. As previously demonstrated for LXR activation [47], the extracts of *H. elongata* and *S. fusiforme* could also favor oligodendrocyte differentiation and enhance (re-)myelination by stimulating cholesterol efflux [42,43] and inhibiting inflammatory responses that hinder re-myelination [43,44,45,46]. Our data showing that OPCs indeed increased the expression of early oligodendrocyte marker O4 upon exposure to the lipid extract of *H. elongata*, but not with the lipid extract of *S. fusiforme*, suggest that the *H. elongata* extract could promote the early stage of oligodendrocyte maturation, as a first step toward (re)myelination. Accordingly, Blanchard et al. [42] demonstrated that the pharmacological promotion of cholesterol transport by cyclodextrin decreases elevated intracellular cholesterol concentrations in APOE4/4 oligodendroglia, promotes myelination, and improves cognitive performance in APOE4 mice. The ability of the *H. elongata* extract to promote cholesterol efflux and oligodendrocyte differentiation—thereby potentially promoting myelination—may have contributed to the observed prevention of cognitive decline in APPswePS1ΔE9 mice.

### 4.8. H. elongata Extract May Reduce the Phosphorylation of Tau and, Thereby, AD-Related Tau Pathology

The *H. elongata* lipid extract, but not the *S. fusiforme* SCF extract, may decrease the AD-associated tau pathology by decreasing the concentration of the AD-associated p-tau variant (Ser202/Thr205). Our data suggest no involvement of the seaweed-derived oxyphytosterol saringosterol nor the *H. elongata*-induced upregulation of desmosterol. The reduction observed in total tau concentrations by the *H. elongata* extract likely reflects the reduction in p-tau concentrations. However, an additional reduction in non-phosphorylated tau—which could disadvantage neuronal stability [95]—cannot be excluded. We demonstrated slightly elevated p-tau concentrations in the forebrain of APPswePS1ΔE9 mice compared to WT mice. However, no significant decline in p-tau in the forebrain of APPswePS1ΔE9 mice was detected upon supplementation with the *H. elongata* lipid extract. Therefore, further research is required to elaborate on the effects of *H. elongata* on tau-related pathology, e.g., in cell lines or animal models displaying more pronounced tau phosphorylation.

We aimed to explore the potential of an *S. fusiforme* extract obtained with supercritical fluid extraction and the potential of the European brown seaweed *H. elongata* in the reduction of AD-related pathology as an alternative to the use of crude *S. fusiforme*. The SCF extraction method can generate extracts with limited to no co-extraction of heavy metals and toxic iAs. Our findings offer an initial glimpse into the potential of this extract in the reduction of AD-related pathology. Additionally, we assessed the potential of a lipid extract of *H. elongata* obtained with the Folch method to assess the potential of *H. elongata*-derived lipids. *H. elongata* shares similarities with *S. fusiforme*, including its saringosterol and fucosterol content and its ability to activate LXRs [28]. However, compared to *S. fusiforme*, *H. elongata* contains low quantities of iAs [27] and, therefore, yields more therapeutic potential than *S. fusiforme*. We demonstrated that the *H. elongata* lipid extract effectively reduced cognitive deterioration and inflammation-related pathology in the APPswePS1ΔE9 mouse model of AD, supporting its potential in the prevention or reduction of AD-related pathology.

A limitation of this study is the small sample size of mice treated with the *S. fusiforme* SCF extract. This discrepancy in group size reduced the statistical power and may explain the lack of statistical significance observed in the trend toward preventing cognitive decline. Furthermore, extending the treatment period of 12 weeks may lead to enhanced outcomes. Nevertheless, this study adequately demonstrated a positive impact of both the *S. fusiforme* SCF extract and the lipid extract of *H. elongata* on inflammation-related pathology, suggesting their potential benefits in inflammation-related (neurodegenerative) conditions. *H. elongata* and *S. fusiforme* are also rich sources of antioxidants and exhibit both antioxidative and hypoglycemic activities [96,97,98,99,100,101]. Consequently, these extracts may hold promise not only for neuroprotection but also for supporting the immune system, cardiovascular health, and blood glucose control, thereby contributing to overall health.

## 5. Conclusions

The lipid extract obtained from *H. elongata* effectively prevented or postponed cognitive decline in the AD mouse model. The SCF extract of *S. fusiforme* demonstrated a similar trend. Underlying mechanisms may involve their ability to mitigate the reactivity of microglia and astrocytes, indicative of potential anti-inflammatory effects, possibly by upregulating the endogenous LXR agonist desmosterol. Despite their LXR-activating capacity, the extracts did not increase hepatic lipid levels. These findings underscore the potential of these extracts in ameliorating the pathophysiology associated with AD and, potentially, other inflammatory or demyelinating conditions. It remains to be investigated whether the positive effect of the extracts on cognition and inflammation is a temporary alleviation or a long-term solution. Hence, more research is necessary to examine the long-term effects of the seaweed extract treatments.

## Figures and Tables

**Table 1:** Nucleotide sequences of primers for Q-PCR on liver tissue.

Gene	Gene Name	Primer Sequence
*ACACA*	Acetyl-CoA Carboxylase Alpha	F: CTCAACAGCGTACAACACCG R: TGGGGATGTTCCCTCTGTTTG
*ACTB*	Actin Beta	F: TTCTTGGGTATGGAATCCTGTGG R: GTCTTTACGGATGTCAACGTCAC
*B2M*	Beta-2-Microglobulin	F: CATGGCTCGCTCGGTGACC R: AATGTGAGGCGGGTGGAACTG
*FASN*	Fatty Acid Synthase	F: GGCCCCTCTGTTAATTGGCT R: GGGATAACAGCACCTTGGTCA
*HPRT1*	Hypoxanthine Phosphoribosyltransferase 1	F: CCTAAGATGAGCGCAAGTTGAA R: CCACAGGACTAGAACACCTGCTAA
*SCD1*	Stearoyl-CoA Desaturase 1	F: GGCCTGTACGGGATCATACTG R: GGTCATGTAGTAGAAAATCCCGAAG
*SDHA*	Succinate Dehydrogenase Complex Flavoprotein Subunit A	F: CTTGAATGAGGCTGACTGTG R: ATCACATAAGCTGGTCCTGT
*SREBF1*	Sterol Regulatory Element Binding Transcription Factor 1	F: GCCATCGACTACATCCGCTT R: CAGGTCCTTCAGTGATTTGCTTT

## Data Availability

Data are available upon request based on the data management policy of the research institute.

## Supplementary Materials

The following supporting information can be downloaded at: https://www.mdpi.com/article/10.3390/nu16111614/s1, Figure S1: Serum and hepatic lipid content upon diet supplementation with *H. elongata* extract and *S. fusiforme* SCF extract, Figure S2: Functional spatial memory of APPswePS1ΔE9 mice at baseline, Figure S3: Sterol profiles after 1 week of supplementation with *H. elongata* extract, Figure S4: F4/80 protein expression in the liver of WT and APPswePS1ΔE9 mice, Figure S5: Tau phosphorylation in differentiated SH-SY5Y cells and in forebrain of APPswePS1ΔE9 and WT mice, Figure S6: PSEN1ΔE9 expression in forebrain and liver of WT and APPswePS1ΔE9 (AD) mice, Table S1: Comparison of APPswePS1ΔE9-related pathways and *H. elongata* lipid extract-affected pathways, Table S2: Ratios of cholesterol precursors, cholesterol metabolites and phytosterols to cholesterol concentrations.

## References

[1] Hardy J., Selkoe D.J. (2002). The amyloid hypothesis of Alzheimer’s disease: Progress and problems on the road to therapeutics. Science.

[2] Blennow K., de Leon M.J., Zetterberg H. (2006). Alzheimer’s disease. Lancet.

[3] Jansen D., Janssen C.I., Vanmierlo T., Dederen P.J., van Rooij D., Zinnhardt B., Nobelen C.L., Janssen A.-L., Hafkemeijer A., Mutsaers M.P. (2012). Cholesterol and synaptic compensatory mechanisms in Alzheimer’s disease mice brain during aging. J. Alzheimers Dis..

[4] Jones L., Holmans P.A., Hamshere M.L., Harold D., Moskvina V., Ivanov D., Pocklington A., Abraham R., Hollingworth P., Sims R. (2010). Genetic evidence implicates the immune system and cholesterol metabolism in the aetiology of Alzheimer’s disease. PLoS ONE.

[5] Kölsch H., Heun R., Jessen F., Popp J., Hentschel F., Maier W., Lütjohann D. (2010). Alterations of cholesterol precursor levels in Alzheimer’s disease. Biochim. Biophys. Acta..

[6] Mulder M. (2009). Sterols in the central nervous system. Curr. Opin. Clin. Nutr. Metab. Care.

[7] Loera-Valencia R., Goikolea J., Parrado-Fernandez C., Merino-Serrais P., Maioli S. (2019). Alterations in cholesterol metabolism as a risk factor for developing Alzheimer’s disease: Potential novel targets for treatment. J. Steroid Biochem. Mol. Biol..

[8] Vanmierlo T., Bloks V.W., van Vark-van der Zee L., Rutten K., Kerksiek A., Friedrichs S., Sijbrands E., Steinbusch H.W., Kuipers F., Lütjohann D. (2010). Alterations in brain cholesterol metabolism in the APPSLxPS1mut mouse, a model for Alzheimer’s disease. J. Alzheimers Dis..

[9] Huang Y., Mucke L. (2012). Alzheimer mechanisms and therapeutic strategies. Cell.

[10] Kloske C.M., Wilcock D.M. (2020). The Important Interface Between Apolipoprotein E and Neuroinflammation in Alzheimer’s Disease. Front. Immunol..

[11] Raulin A.C., Doss S.V., Trottier Z.A., Ikezu T.C., Bu G., Liu C.C. (2022). ApoE in Alzheimer’s disease: Pathophysiology and therapeutic strategies. Mol. Neurodegener..

[12] Zelcer N., Tontonoz P. (2006). Liver X receptors as integrators of metabolic and inflammatory signaling. J. Clin. Investig..

[13] Xu X., Xiao X., Yan Y., Zhang T. (2021). Activation of liver X receptors prevents emotional and cognitive dysfunction by suppressing microglial M1-polarization and restoring synaptic plasticity in the hippocampus of mice. Brain Behav. Immun..

[14] Moutinho M., Landreth G.E. (2017). Therapeutic potential of nuclear receptor agonists in Alzheimer’s disease. J. Lipid Res..

[15] Lanfranco M.F., Ng C.A., Rebeck G.W. (2020). ApoE Lipidation as a Therapeutic Target in Alzheimer’s Disease. Int. J. Mol. Sci..

[16] Mouzat K., Chudinova A., Polge A., Kantar J., Camu W., Raoul C., Lumbroso S. (2019). Regulation of Brain Cholesterol: What Role Do Liver X Receptors Play in Neurodegenerative Diseases?. Int. J. Mol. Sci..

[17] Donkin J.J., Stukas S., Hirsch-Reinshagen V., Namjoshi D., Wilkinson A., May S., Chan J., Fan J., Collins J., Wellington C.L. (2010). ATP-binding cassette transporter A1 mediates the beneficial effects of the liver X receptor agonist GW3965 on object recognition memory and amyloid burden in amyloid precursor protein/presenilin 1 mice. J. Biol. Chem..

[18] Jiang Q., Lee C.D., Mandrekar S., Wilkinson B., Cramer P., Zelcer N., Mann K., Lamb B., Willson T.M., Collins J.L. (2008). ApoE promotes the proteolytic degradation of Abeta. Neuron.

[19] Vanmierlo T., Rutten K., Dederen J., Bloks V.W., van Vark-van der Zee L.C., Kuipers F., Kiliaan A., Blokland A., Sijbrands E.J., Steinbusch H. (2011). Liver X receptor activation restores memory in aged AD mice without reducing amyloid. Neurobiol. Aging.

[20] Riddell D.R., Zhou H., Comery T.A., Kouranova E., Lo C.F., Warwick H.K., Ring R.H., Kirksey Y., Aschmies S., Xu J. (2007). The LXR agonist TO901317 selectively lowers hippocampal Abeta42 and improves memory in the Tg2576 mouse model of Alzheimer’s disease. Mol. Cell Neurosci..

[21] Grefhorst A., Elzinga B.M., Voshol P.J., Plo¨sch T., Kok T., Bloks V.W., van der Sluijs F.H., Havekes L.M., Romijn J.A., Verkade H.J. (2002). Stimulation of lipogenesis by pharmacological activation of the liver X receptor leads to production of large, triglyceride-rich very low density lipoprotein particles. J. Biol. Chem..

[22] Repa J.J., Liang G., Ou J., Bashmakov Y., Lobaccaro J.M., Shimomura I., Shan B., Brown M.S., Goldstein J.L., Mangelsdorf D.J. (2000). Regulation of mouse sterol regulatory element-binding protein-1c gene (SREBP-1c) by oxysterol receptors, LXRalpha and LXRbeta. Genes. Dev..

[23] Schultz J.R., Tu H., Luk A., Repa J.J., Medina J.C., Li L., Schwendner S., Wang S., Thoolen M., Mangelsdorf D.J. (2000). Role of LXRs in control of lipogenesis. Genes. Dev..

[24] Martens N., Schepers M., Zhan N., Leijten F., Voortman G., Tiane A., Rombaut B., Poisquet J., van de Sande N., Kerksiek A. (2021). 24(S)-Saringosterol Prevents Cognitive Decline in a Mouse Model for Alzheimer’s Disease. Mar. Drugs..

[25] Bogie J., Hoeks C., Schepers M., Tiane A., Cuypers A., Leijten F., Chintapakorn Y., Suttiyut T., Pornpakakul S., Struik D. (2019). Dietary *Sargassum fusiforme* improves memory and reduces amyloid plaque load in an Alzheimer’s disease mouse model. Sci. Rep..

[26] Rose M., Lewis J., Langford N., Baxter M., Origgi S., Barber M., MacBain H., Thomas K. (2007). Arsenic in seaweed—Forms, concentration and dietary exposure. Food Chem. Toxicol..

[27] Besada V., Andrade J.M., Schultze F., González J.J. (2009). Heavy metals in edible seaweeds commercialised for human consumption. J. Mar. Syst..

[28] Martens N., Zhan N., Voortman G., Leijten F.P., van Rheenen C., van Leerdam S., Geng X., Huybrechts M., Liu H., Jonker J.W. (2023). Activation of Liver X Receptors and Peroxisome Proliferator-Activated Receptors by Lipid Extracts of Brown Seaweeds: A Potential Application in Alzheimer’s Disease?. Nutrients.

[29] Zwarts I., van Zutphen T., Kruit J.K., Liu W., Oosterveer M.H., Verkade H.J., Uhlenhaut N.H., Jonker J.W. (2019). Identification of the fructose transporter GLUT5 (SLC2A5) as a novel target of nuclear receptor, L.X.R. Sci. Rep..

[30] Dixon W.J. (1950). Analysis of extreme values. Ann. Math. Stat..

[31] Dixon W.J. (1951). Ratios involving extreme values. Ann. Math. Stat..

[32] Bligh E.G., Dyer W.J. (1959). A rapid method of total lipid extraction and purification. Can. J. Biochem. Physiol..

[33] Fedoseienko A., Wijers M., Wolters J.C., Dekker D., Smit M., Huijkman N., Kloosterhuis N., Klug H., Schepers A., van Dijk K.W. (2018). The COMMD Family Regulates Plasma LDL Levels and Attenuates Atherosclerosis Through Stabilizing the CCC Complex in Endosomal LDLR Trafficking. Circ. Res..

[34] Larsen L.E., Boogert M.A.v.D., Rios-Ocampo W.A., Jansen J.C., Conlon D., Chong P.L., Levels J.H.M., Eilers R.E., Sachdev V.V., Zelcer N. (2022). Defective Lipid Droplet-Lysosome Interaction Causes Fatty Liver Disease as Evidenced by Human Mutations in TMEM199 and CCDC115. Cell Mol. Gastroenterol. Hepatol..

[35] Arganda-Carreras I., Kaynig V., Rueden C., Eliceiri K.W., Schindelin J., Cardona A., Seung H.S. (2017). Trainable Weka Segmentation: A machine learning tool for microscopy pixel classification. Bioinformatics.

[36] Adorni M.P., Papotti B., Borghi M.O., Raschi E., Zimetti F., Bernini F., Meroni P.L., Ronda N. (2023). Effect of the JAK/STAT Inhibitor Tofacitinib on Macrophage Cholesterol Metabolism. Int. J. Mol. Sci..

[37] Turri M., Conti E., Pavanello C., Gastoldi F., Palumbo M., Bernini F., Aprea V., Re F., Barbiroli A., Emide D. (2023). Plasma and cerebrospinal fluid cholesterol esterification is hampered in Alzheimer’s disease. Alzheimers Res. Ther..

[38] Tiane A., Schepers M., Riemens R., Rombaut B., Vandormael P., Somers V., Prickaerts J., Hellings N., Hove D.v.D., Vanmierlo T. (2021). DNA methylation regulates the expression of the negative transcriptional regulators ID2 and ID4 during OPC differentiation. Cell Mol. Life Sci..

[39] Love M.I., Huber W., Anders S. (2014). Moderated estimation of fold change and dispersion for RNA-seq data with DESeq2. Genome Biol..

[40] Lütjohann D., Brzezinka A., Barth E., Abramowski D., Staufenbiel M., von Bergmann K., Beyreuther K., Multhaup G., Bayer T.A. (2002). Profile of cholesterol-related sterols in aged amyloid precursor protein transgenic mouse brain. J. Lipid Res..

[41] Akkerman S., Prickaerts J., Steinbusch H.W., Blokland A. (2012). Object recognition testing: Statistical considerations. Behav. Brain Res..

[42] Blanchard J.W., Akay L.A., Davila-Velderrain J., von Maydell D., Mathys H., Davidson S.M., Effenberger A., Chen C.Y., Maner-Smith K., Hajjar I. (2022). APOE4 impairs myelination via cholesterol dysregulation in oligodendrocytes. Nature.

[43] Cantuti-Castelvetri L., Fitzner D., Bosch-Queralt M., Weil M.-T., Su M., Sen P., Ruhwedel T., Mitkovski M., Trendelenburg G., Lütjohann D. (2018). Defective cholesterol clearance limits remyelination in the aged central nervous system. Science.

[44] Hindinger C., Hinton D.R., Kirwin S.J., Atkinson R.D., Burnett M.E., Bergmann C.C., Stohlman S.A. (2006). Liver X receptor activation decreases the severity of experimental autoimmune encephalomyelitis. J. Neurosci. Res..

[45] Cui G., Qin X., Wu L., Zhang Y., Sheng X., Yu Q., Sheng H., Xi B., Zhang J.Z., Zang Y.Q. (2011). Liver X receptor (LXR) mediates negative regulation of mouse and human Th17 differentiation. J. Clin. Investig..

[46] Berghoff S.A., Spieth L., Sun T., Hosang L., Schlaphoff L., Depp C., Düking T., Winchenbach J., Neuber J., Ewers D. (2021). Microglia facilitate repair of demyelinated lesions via post-squalene sterol synthesis. Nat. Neurosci..

[47] Meffre D., Shackleford G., Hichor M., Gorgievski V., Tzavara E.T., Trousson A., Ghoumari A.M., Deboux C., Oumesmar B.N., Liere P. (2015). Liver X receptors alpha and beta promote myelination and remyelination in the cerebellum. Proc. Natl. Acad. Sci. USA.

[48] Depp C., Sun T., Sasmita A.O., Spieth L., Berghoff S.A., Nazarenko T., Overhoff K., Steixner-Kumar A.A., Subramanian S., Arinrad S. (2023). Myelin dysfunction drives amyloid-β deposition in models of Alzheimer’s disease. Nature.

[49] van der Kant R., Goldstein L.S.B., Ossenkoppele R. (2020). Amyloid-β-independent regulators of tau pathology in Alzheimer disease. Nat. Rev. Neurosci..

[50] van der Kant R., Langness V.F., Herrera C.M., Williams D.A., Fong L.K., Leestemaker Y., Steenvoorden E., Rynearson K.D., Brouwers J.F., Helms J.B. (2019). Cholesterol metabolism is a druggable axis that independently regulates tau and amyloid-β in iPSC-derived Alzheimer’s disease neurons. Cell Stem Cell.

[51] Litvinchuk A., Suh J.H., Guo J.L., Lin K., Davis S.S., Bien-Ly N., Tycksen E., Tabor G.T., Serrano J.R., Manis M. (2024). Amelioration of Tau and ApoE4-linked glial lipid accumulation and neurodegeneration with an LXR agonist. Neuron.

[52] Snowdon D.A. (1997). Aging and Alzheimer’s disease: Lessons from the Nun Study. Gerontologist.

[53] Green K.N., Steffan J.S., Martinez-Coria H., Sun X., Schreiber S.S., Thompson L.M., LaFerla F.M. (2008). Nicotinamide restores cognition in Alzheimer’s disease transgenic mice via a mechanism involving sirtuin inhibition and selective reduction of Thr231-phosphotau. J. Neurosci..

[54] Ikeda I., Tanaka K., Sugano M., Vahouny G.V., Gallo L.L. (1988). Inhibition of cholesterol absorption in rats by plant sterols. J. Lipid Res..

[55] Yu L., York J., von Bergmann K., Lutjohann D., Cohen J.C., Hobbs H.H. (2003). Stimulation of cholesterol excretion by the liver X receptor agonist requires ATP-binding cassette transporters G5 and G8. J. Biol. Chem..

[56] Radhakrishnan A., Sun L.P., Kwon H.J., Brown M.S., Goldstein J.L. (2004). Direct binding of cholesterol to the purified membrane region of SCAP: Mechanism for a sterol-sensing domain. Mol. Cell..

[57] Yang C., McDonald J.G., Patel A., Zhang Y., Umetani M., Xu F., Westover E.J., Covey D.F., Mangelsdorf D.J., Cohen J.C. (2006). Sterol intermediates from cholesterol biosynthetic pathway as liver X receptor ligands. J. Biol. Chem..

[58] Gupta S., Pandak W.M., Hylemon P.B. (2002). LXR alpha is the dominant regulator of CYP7A1 transcription. Biochem. Biophys. Res. Commun..

[59] Chiang J.Y.L., Ferrell J.M. (2020). Up to date on cholesterol 7 alpha-hydroxylase (CYP7A1) in bile acid synthesis. Liver Res..

[60] Uppal H., Saini S.P., Moschetta A., Mu Y., Zhou J., Gong H., Zhai Y., Ren S., Michalopoulos G.K., Mangelsdorf D.J. (2007). Activation of LXRs prevents bile acid toxicity and cholestasis in female mice. Hepatology.

[61] Feringa F.M., van der Kant R. (2021). Cholesterol and Alzheimer’s Disease; From Risk Genes to Pathological Effects. Front. Aging Neurosci..

[62] Mitsche M.A., McDonald J.G., Hobbs H.H., Cohen J.C. (2015). Flux analysis of cholesterol biosynthesis in vivo reveals multiple tissue and cell-type specific pathways. Elife.

[63] Fernández C., Suárez Y., Ferruelo A.J., Gómez-Coronado D., Lasunción M.A. (2002). Inhibition of cholesterol biosynthesis by Delta22-unsaturated phytosterols via competitive inhibition of sterol Delta24-reductase in mammalian cells. Biochem. J..

[64] Zerenturk E.J., Kristiana I., Gill S., Brown A.J. (2012). The endogenous regulator 24(S),25-epoxycholesterol inhibits cholesterol synthesis at DHCR24 (Seladin-1). Biochim. Biophys. Acta..

[65] Pfeifer T., Buchebner M., Chandak P.G., Patankar J., Kratzer A., Obrowsky S., Rechberger G.N., Kadam R.S., Kompella U.B., Kostner G.M. (2011). Synthetic LXR agonist suppresses endogenous cholesterol biosynthesis and efficiently lowers plasma cholesterol. Curr. Pharm. Biotechnol..

[66] Daimiel L.A., Fernandez-Suarez M.E., Rodriguez-Acebes S., Crespo L., Lasuncion M.A., Gomez-Coronado D., Martinez-Botas J. (2012). Promoter analysis of the DHCR24 (3β-hydroxysterol Δ(24)-reductase) gene: Characterization of SREBP (sterol-regulatory-element-binding protein)-mediated activation. Biosci. Rep..

[67] Zerenturk E.J., Sharpe L.J., Brown A.J. (2012). Sterols regulate 3β-hydroxysterol Δ24-reductase (DHCR24) via dual sterol regulatory elements: Cooperative induction of key enzymes in lipid synthesis by Sterol Regulatory Element Binding Proteins. Biochim. Biophys. Acta..

[68] Sander P., Hamann H., Drögemüller C., Kashkevich K., Schiebel K., Leeb T. (2005). Bovine prion protein gene (PRNP) promoter polymorphisms modulate PRNP expression and may be responsible for differences in bovine spongiform encephalopathy susceptibility. J. Biol. Chem..

[69] Chen L., van den Munckhof I.C.L., Schraa K., Ter Horst R., Koehorst M., Van Faassen M., Van Der Ley C., Doestzada M., Zhernakova D.V., Kurilshikov A. (2020). Genetic and Microbial Associations to Plasma and Fecal Bile Acids in Obesity Relate to Plasma Lipids and Liver Fat Content. Cell Rep..

[70] Biallosterski B., de Wachter S., van Koeveringe G., van Kerrebroeck P., de Vente J., Mulder M., Gillespie J. (2010). Changes in bladder innervation in a mouse model of Alzheimer’s disease. J. Chem. Neuroanat..

[71] Kaur H., Seeger D., Golovko S., Golovko M., Combs C.K. (2021). Liver Bile Acid Changes in Mouse Models of Alzheimer’s Disease. Int. J. Mol. Sci..

[72] Nho K., Kueider-Paisley A., MahmoudianDehkordi S., Arnold M., Risacher S.L., Louie G., Blach C., Baillie R., Han X., Kastenmueller G. (2019). Altered bile acid profile in mild cognitive impairment and Alzheimer’s disease: Relationship to neuroimaging and CSF biomarkers. Alzheimers Dement..

[73] Guo Y., Wang Q., Chen S., Xu C. (2021). Functions of amyloid precursor protein in metabolic diseases. Metabolism.

[74] Di Benedetto G., Burgaletto C., Bellanca C.M., Munafò A., Bernardini R., Cantarella G. (2022). Role of Microglia and Astrocytes in Alzheimer’s Disease: From Neuroinflammation to Ca(2+) Homeostasis Dysregulation. Cells.

[75] Olsthoorn S.E.M., Wang X., Tillema B., Vanmierlo T., Kraan S., Leenen P.J.M., Mulder M.T. (2021). Brown Seaweed Food Supplementation: Effects on Allergy and Inflammation and Its Consequences. Nutrients.

[76] Fiala M., Liu Q.N., Sayre J., Pop V., Brahmandam V., Graves M.C., Vinters H.V. (2002). Cyclooxygenase-2-positive macrophages infiltrate the Alzheimer’s disease brain and damage the blood-brain barrier. Eur. J. Clin. Investig..

[77] Heneka M.T., Rodríguez J.J., Verkhratsky A. (2010). Neuroglia in neurodegeneration. Brain Res. Rev..

[78] Heller C., Foiani M.S., Moore K., Convery R., Bocchetta M., Neason M., Cash D.M., Thomas D., Greaves C.V., Woollacott I.O. (2020). Plasma glial fibrillary acidic protein is raised in progranulin-associated frontotemporal dementia. J. Neurol. Neurosurg. Psychiatry..

[79] Abdelhak A., Foschi M., Abu-Rumeileh S., Yue J.K., D’anna L., Huss A., Oeckl P., Ludolph A.C., Kuhle J., Petzold A. (2022). Blood GFAP as an emerging biomarker in brain and spinal cord disorders. Nat. Rev. Neurol..

[80] Dvorak F., Haberer I., Sitzer M., Foerch C. (2009). Characterisation of the diagnostic window of serum glial fibrillary acidic protein for the differentiation of intracerebral haemorrhage and ischaemic stroke. Cerebrovasc. Dis..

[81] Shir D., Graff-Radford J., Hofrenning E.I., Lesnick T.G., Przybelski S.A., Lowe V.J., Knopman D.S., Petersen R.C., Jack C.R., Vemuri P. (2022). Association of plasma glial fibrillary acidic protein (GFAP) with neuroimaging of Alzheimer’s disease and vascular pathology. Alzheimers Dement.

[82] Cicognola C., Janelidze S., Hertze J., Zetterberg H., Blennow K., Mattsson-Carlgren N., Hansson O. (2021). Plasma glial fibrillary acidic protein detects Alzheimer pathology and predicts future conversion to Alzheimer dementia in patients with mild cognitive impairment. Alzheimers Res. Ther..

[83] Monterey M.D., Wei H., Wu X., Wu J.Q. (2021). The Many Faces of Astrocytes in Alzheimer’s Disease. Front. Neurol..

[84] Frost G.R., Li Y.M. (2017). The role of astrocytes in amyloid production and Alzheimer’s disease. Open Biol..

[85] Spann N.J., Garmire L.X., McDonald J.G., Myers D.S., Milne S.B., Shibata N., Reichart D., Fox J.N., Shaked I., Heudobler D. (2012). Regulated accumulation of desmosterol integrates macrophage lipid metabolism and inflammatory responses. Cell.

[86] Körner A., Zhou E., Müller C., Mohammed Y., Herceg S., Bracher F., Rensen P.C.N., Wang Y., Mirakaj V., Giera M. (2019). Inhibition of Δ24-dehydrocholesterol reductase activates pro-resolving lipid mediator biosynthesis and inflammation resolution. Proc. Natl. Acad. Sci. USA.

[87] Zhou E., Ge X., Nakashima H., Li R., van der Zande H.J.P., Liu C., Li Z., Müller C., Bracher F., Mohammed Y. (2023). Inhibition of DHCR24 activates LXRα to ameliorate hepatic steatosis and inflammation. EMBO Mol. Med..

[88] Sadigh-Eteghad S., Majdi A., Mahmoudi J., Golzari S.E.J., Talebi M. (2016). Astrocytic and microglial nicotinic acetylcholine receptors: An overlooked issue in Alzheimer’s disease. J. Neural. Transm..

[89] Horkowitz A.P., Schwartz A., Alvarez C.A., Herrera E.B., Thoman M.L., Chatfield D.A., Osborn K.G., Feuer R., George U.Z., Phillips J.A. (2020). Acetylcholine Regulates Pulmonary Pathology during Viral Infection and Recovery. Immunotargets Ther..

[90] Guo Y., Ma X., Li P., Dong S., Huang X., Ren X., Yuan L. (2020). High-fat diet induced discrepant peripheral and central nervous systems insulin resistance in APPswe/PS1dE9 and wild-type C57BL/6J mice. Aging.

[91] Müller L., Guerra N.P., Stenzel J., Rühlmann C., Lindner T., Krause B.J., Vollmar B., Teipel S., Kuhla A. (2021). Long-Term Caloric Restriction Attenuates β-Amyloid Neuropathology and Is Accompanied by Autophagy in APPswe/PS1delta9 Mice. Nutrients.

[92] Patel N.V., Gordon M.N., Connor K.E., Good R.A., Engelman R.W., Mason J., Morgan D.G., Morgan T.E., Finch C.E. (2005). Caloric restriction attenuates Aβ-deposition in Alzheimer transgenic models. Neurobiol. Aging.

[93] Halagappa V.K.M., Guo Z., Pearson M., Matsuoka Y., Cutler R.G., LaFerla F.M., Mattson M.P. (2007). Intermittent fasting and caloric restriction ameliorate age-related behavioral deficits in the triple-transgenic mouse model of Alzheimer’s disease. Neurobiol. Dis..

[94] Zhang Y., Repa J.J., Gauthier K., Mangelsdorf D.J. (2001). Regulation of lipoprotein lipase by the oxysterol receptors, LXRalpha and LXRbeta. J. Biol. Chem..

[95] Feinstein S.C., Wilson L. (2005). Inability of tau to properly regulate neuronal microtubule dynamics: A loss-of-function mechanism by which tau might mediate neuronal cell death. Biochim. Biophys. Acta (BBA)-Mol. Basis Dis..

[96] Lamela M., Anca J., Villar R., Otero J., Calleja J.M. (1989). Hypoglycemic activity of several seaweed extracts. J. Ethnopharmacol..

[97] Moreira A.R., Garcimartín A., Bastida S., Jiménez-Escrig A., Rupérez P., Green B.D., Rafferty E., Sánchez-Muniz F.J., Benedí J. (2014). Effects of Undaria pinnatifida, *Himanthalia elongata* and Porphyra umbilicalis extracts on in vitro α-glucosidase activity and glucose diffusion. Nutr. Hosp..

[98] Ilyas Z., Ali Redha A., Wu Y.S., Ozeer F.Z., Aluko R.E. (2023). Nutritional and Health Benefits of the Brown Seaweed *Himanthalia elongata*. Plant Foods Hum. Nutr..

[99] Fernández-Segovia I., Lerma-García M.J., Fuentes A., Barat J.M. (2018). Characterization of Spanish powdered seaweeds: Composition, antioxidant capacity and technological properties. Food Res. Int..

[100] Vanmierlo T., Schepers M., Martens N., Tiane A., Vanbrabant K., Liu H.-B., Lütjohann D., Mulder M. (2020). Edible seaweed-derived constituents: An undisclosed source of neuroprotective compounds. Neural Regen. Res..

[101] Zhang R., Zhang X., Tang Y., Mao J. (2020). Composition, isolation, purification and biological activities of *Sargassum fusiforme* polysaccharides: A review. Carbohydr. Polym..

