# Supplementary material for: Supplementation of Seaweed Extracts to the Diet Reduces Symptoms of Alzheimer’s Disease in the APPswePS1ΔE9 Mouse Model
Source: Nutrients. 2024 May 25;16(11):1614. doi: 10.3390/nu16111614 (PMC11174572; doi:10.3390/nu16111614)
Supplement: Supplementary file 1 [file nutrients-16-01614-s001.zip › nutrients-2995240-supplementary.pdf]

nutrients

Supplementary Materials

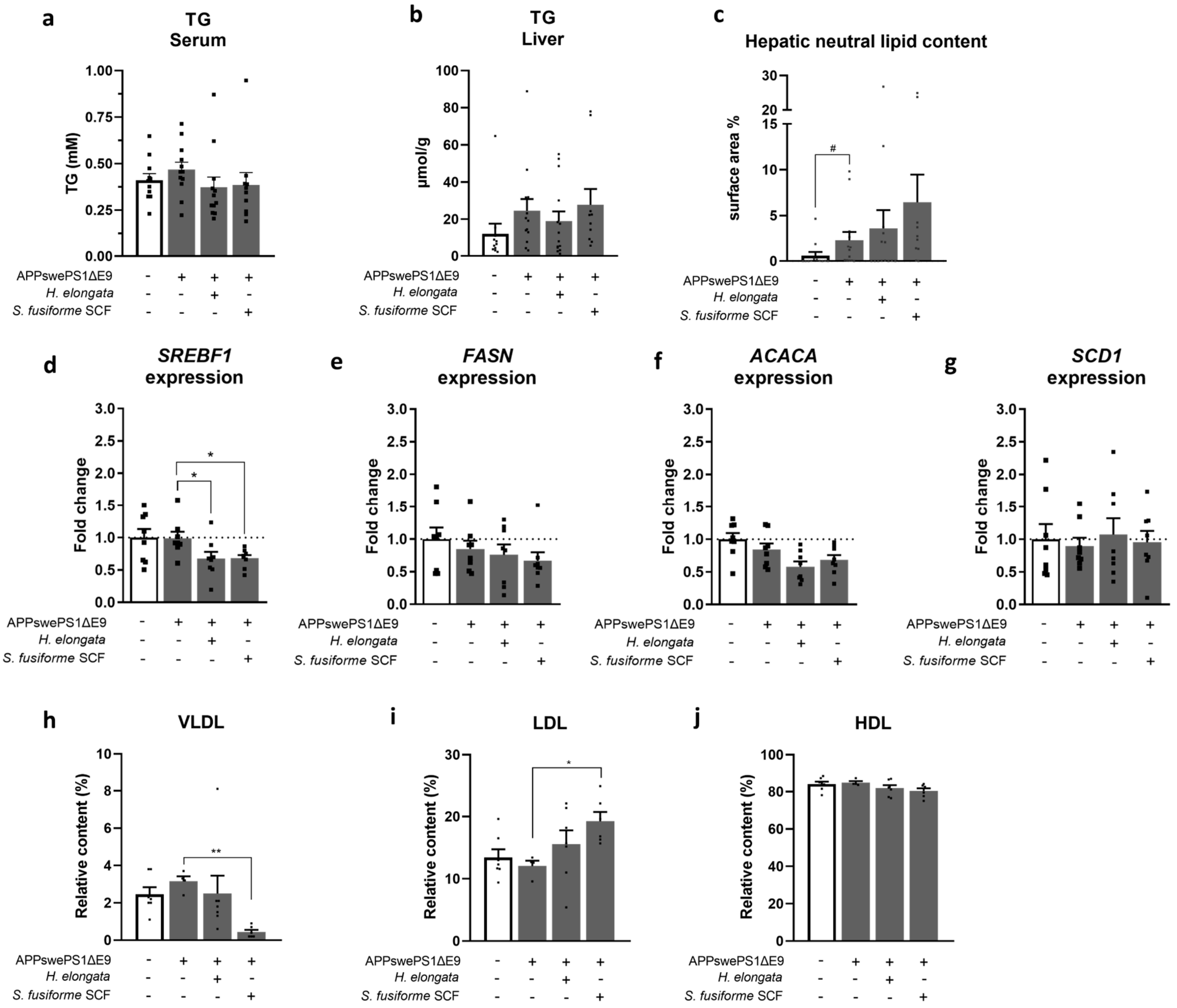

Figure S1. Serum and hepatic lipid content upon diet supplementation with *H. elongata* extract and *S. fusiforme* SCF extract. Serum and liver triglyceride (TG) concentrations (**a**,**b**), and hepatic neutral lipid content presented as surface area percentage of Oil Red O staining (**c**) are presented (n = 10–14 per group). The expression of SREBF1 (**d**), its target genes FASN (**e**), ACACA (**f**), and SCD1 (**g**) in the liver was normalized to the stable housekeeping genes (ACTB, B2M, HPRT1, and SDHA) and expressed as fold change relative to the expression in vehicle-treated WT mice (n = 8 per group). The cholesterol concentrations in VLDL (**h**), LDL (**i**), and HDL (**j**) relative to total cholesterol content of the lipoprotein fractions are presented (n = 4–9 per group). Data are presented as mean ± SEM. Differences between vehicle-treated APPswePS1ΔE9 and WT mice were analyzed with an unpaired t-test (figures d, f, and g) or a Mann Whitney U test (figures **a**–**c**,**e**,**h**–**j**) (# $p < 0.05$); Treatment effects in APPswePS1ΔE9 mice were analyzed with an one-way ANOVA (figures **d**,**f**,**g**) or a Kruskal-Wallis test (figures **a**–**c**,**e**,**h**–**j**) (with Dunnett's/Dunn's multiple comparisons test) (* $p < 0.05$, ** $p < 0.01$).

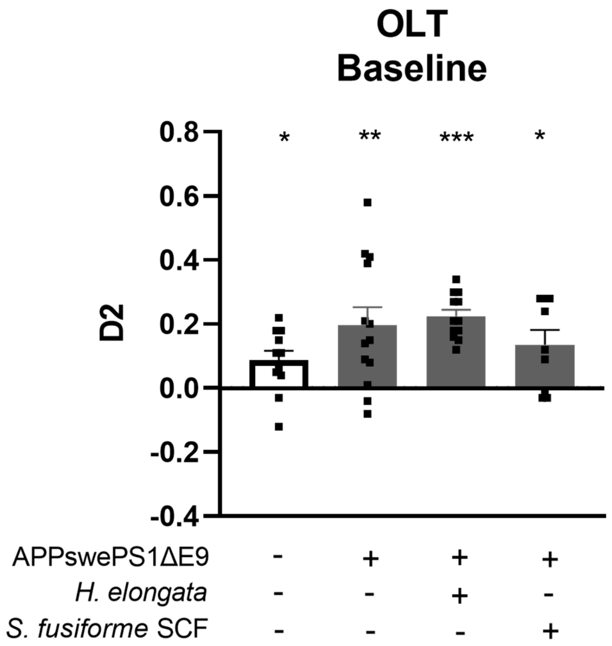

Figure S2. Functional spatial memory of APPswePS1ΔE9 mice at baseline. The cognitive performance was determined using an object location task (OLT) with an inter-trial interval of 4 hours. Bars represent mean ± SEM (n = 9–13 per group). Data were analyzed with a one-sample t-test compared to 0. D2 values relative to 0: * $p \leq 0.05$, ** $p \leq 0.01$, *** $p \leq 0.001$.

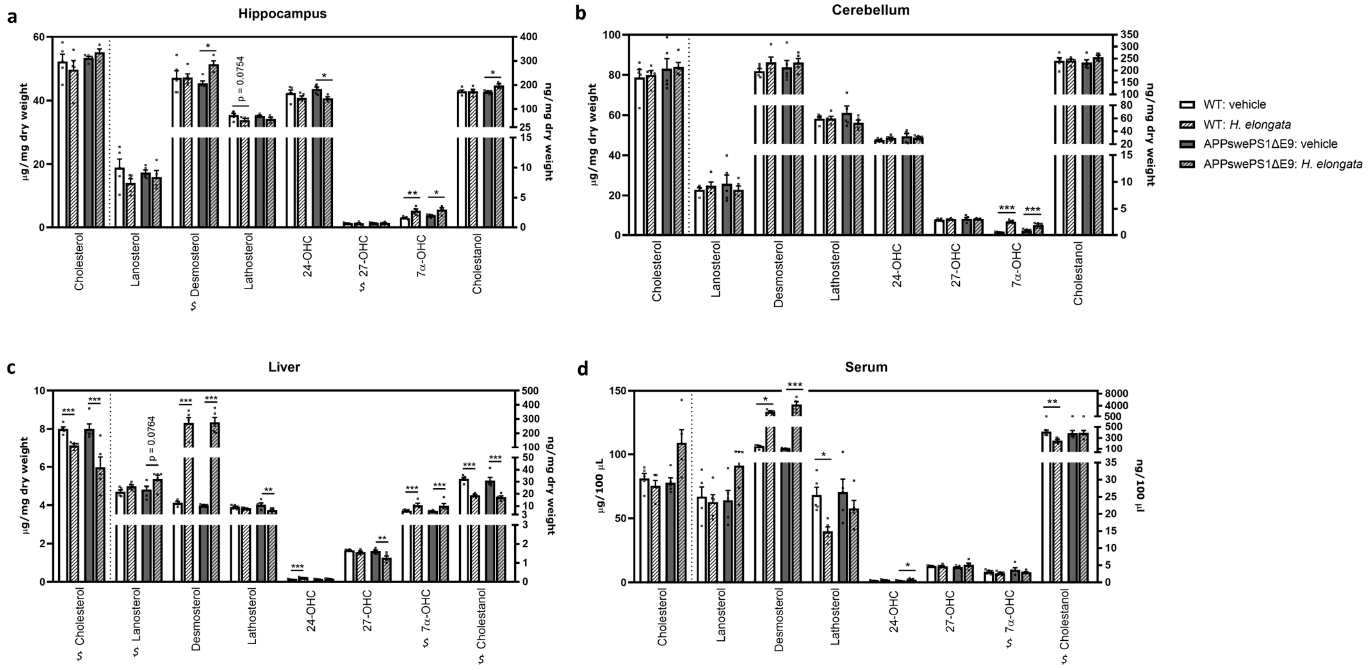

Figure S3. Sterol profiles after 1 week of supplementation with *H. elongata* extract. The concentrations of cholesterol in the hippocampus (**a**), cerebellum (**b**), liver (**c**) and serum (**d**) of WT and APPswePS1ΔE9 mice are presented on the left Y-axis whereas the concentrations of cholesterol precursors (lanosterol, desmosterol, and lathosterol), cholesterol metabolites (24-OHC, 27-OHC, 7α-OHC, and cholestanol) and fucosterol are presented on the right Y-axis. Data are presented as mean ± SEM (n = 4–5 per group) and analyzed with two-way ANOVA (post-hoc: Sidak), prior to which the data indicated with a '$' went through a rank transformation. * $p \leq 0.05$, ** $p \leq 0.01$, *** $p \leq 0.001$.

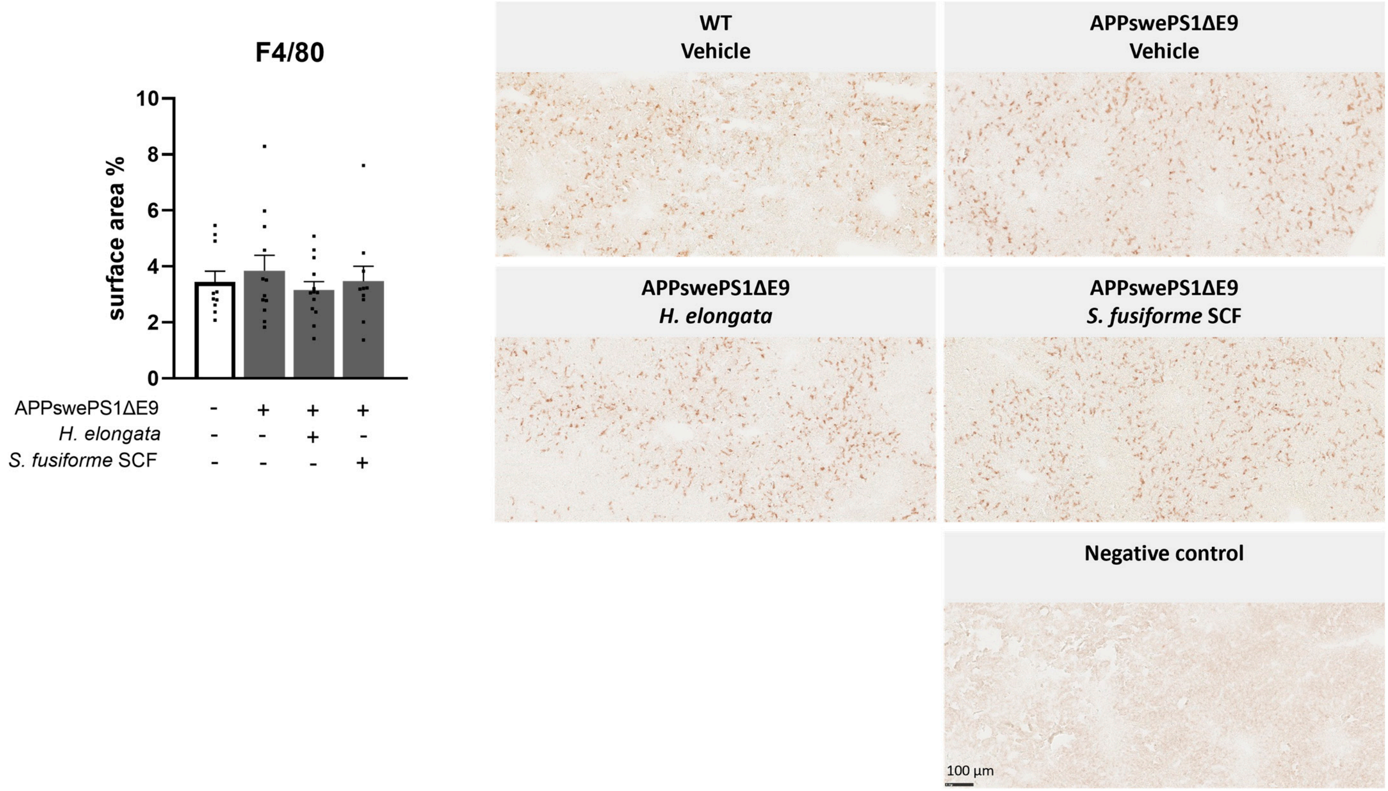

Figure S4. F4/80 protein expression in the liver of WT and APPswePS1ΔE9 mice. The quantification of the immunohistochemical staining of macrophage marker F4/80 in the liver of WT and APPswePS1ΔE9 mice are presented as relative surface area. Representative images of the F4/80 staining are presented. Data are represented as mean ± SEM (n = 10–13 per group, 3 image of 1 slide per animal). Differences between vehicle-treated APPswePS1ΔE9 and WT mice were analyzed with a Mann-Whitney U test (no statistically significant difference detected); Treatment effects in APPswePS1ΔE9 mice were analyzed with a Kruskal-Wallis test (with Dunn's multiple comparisons test) (no statistically significant differences detected).

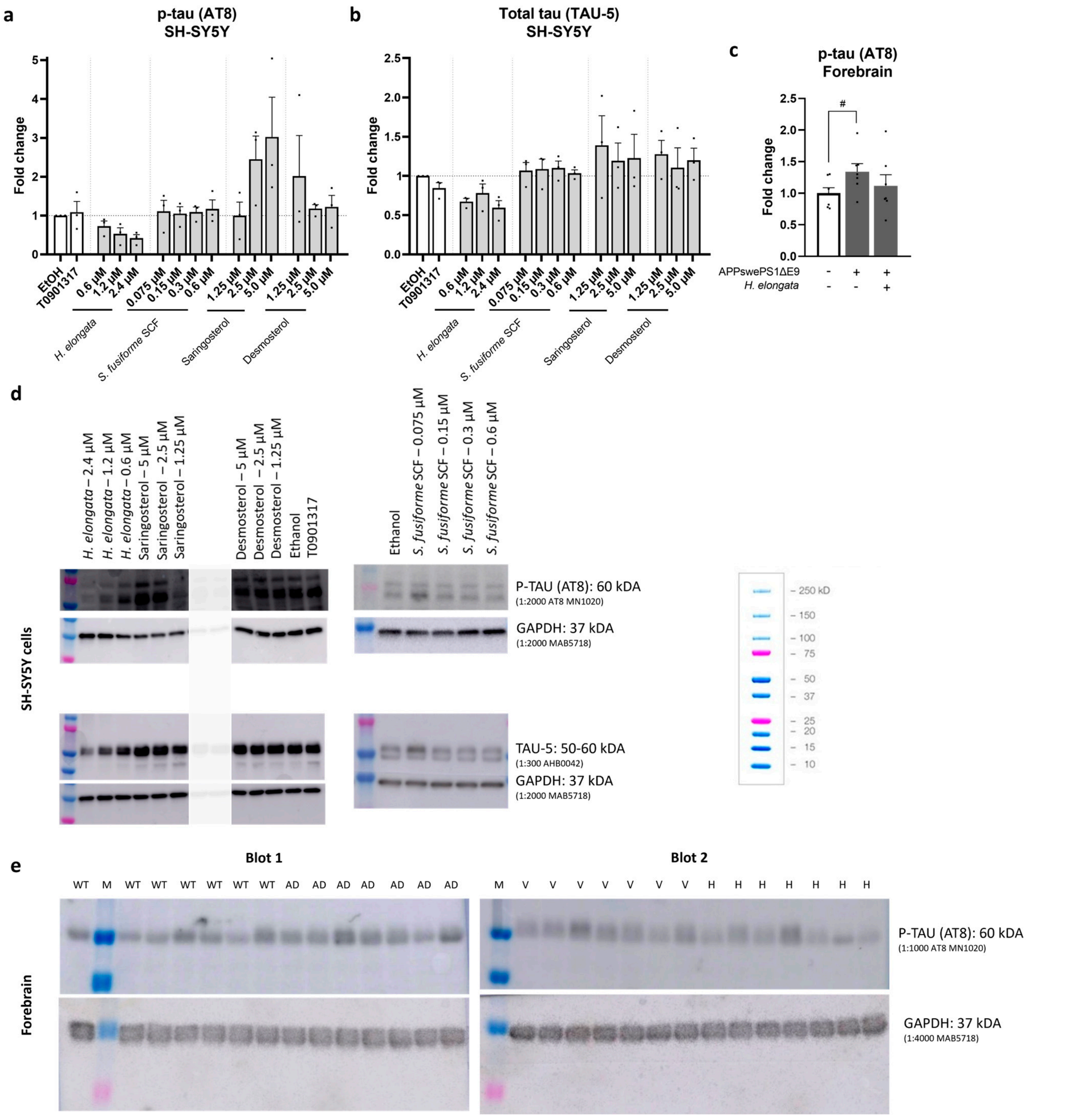

Figure S5. Tau phosphorylation in differentiated SH-SY5Y cells and in forebrain of APPswePS1ΔE9 and WT mice. Phosphorylated tau (p-tau; Ser202, Thr205 (AT8)) (**a**) and total tau (TAU-5) (**b**) were quantified using western blot in differentiated SH-SY5Y cells incubated with H. elongata lipid extract, S fusiforme SCF extract, saringosterol, desmosterol, or LXR agonist T0901317 (1 µM) (n = 3 of three independent experiments). The extracts were added based on their saringosterol concentration (on X-axis). Phosphorylated Tau (p-tau; Ser202, Thr205; AT8) was also quantified in the forebrain of WT and APPswePS1ΔE9 mice after 12 weeks of supplementation with H. elongata lipid extract (**c**) (n = 7 per group). The p-tau and total tau levels were normalized to GAPDH and expressed as fold change relative to ethanol-exposed SH-SY5Y cells or vehicle-treated WT mice. Representative images of western blots with protein samples of differentiated SH-SY5Y cells—which were incubated with H. elongata lipid extract, S fusiforme SCF extract, saringosterol, desmosterol, or LXR agonist T0901317 (1 µM)—stained for phosphorylated tau (p-tau; Ser202, Thr205 (AT8)) and total tau (TAU-5) are presented in figure d. Presentative images of western blots with protein samples of forebrains

of vehicle-treated WT and APPswePS1ΔE9 (AD) mice (blot 1) and vehicle-treated (V) and H. elongata-treated (H) APPswePS1ΔE9 mice (blot 2) stained for phosphorylated tau (p-tau; Ser202, Thr205 (AT8)) are presented in figure e. Data are presented as mean ± SEM. For data in figure c, differences between vehicle-treated APPswePS1ΔE9 and WT mice were analyzed with an unpaired t-test (# $p <$ 0.05); the difference between vehicle-treated and extract-treated APPswePS1ΔE9 mice was analyzed with an unpaired *t*-test, but was not statistically significant.

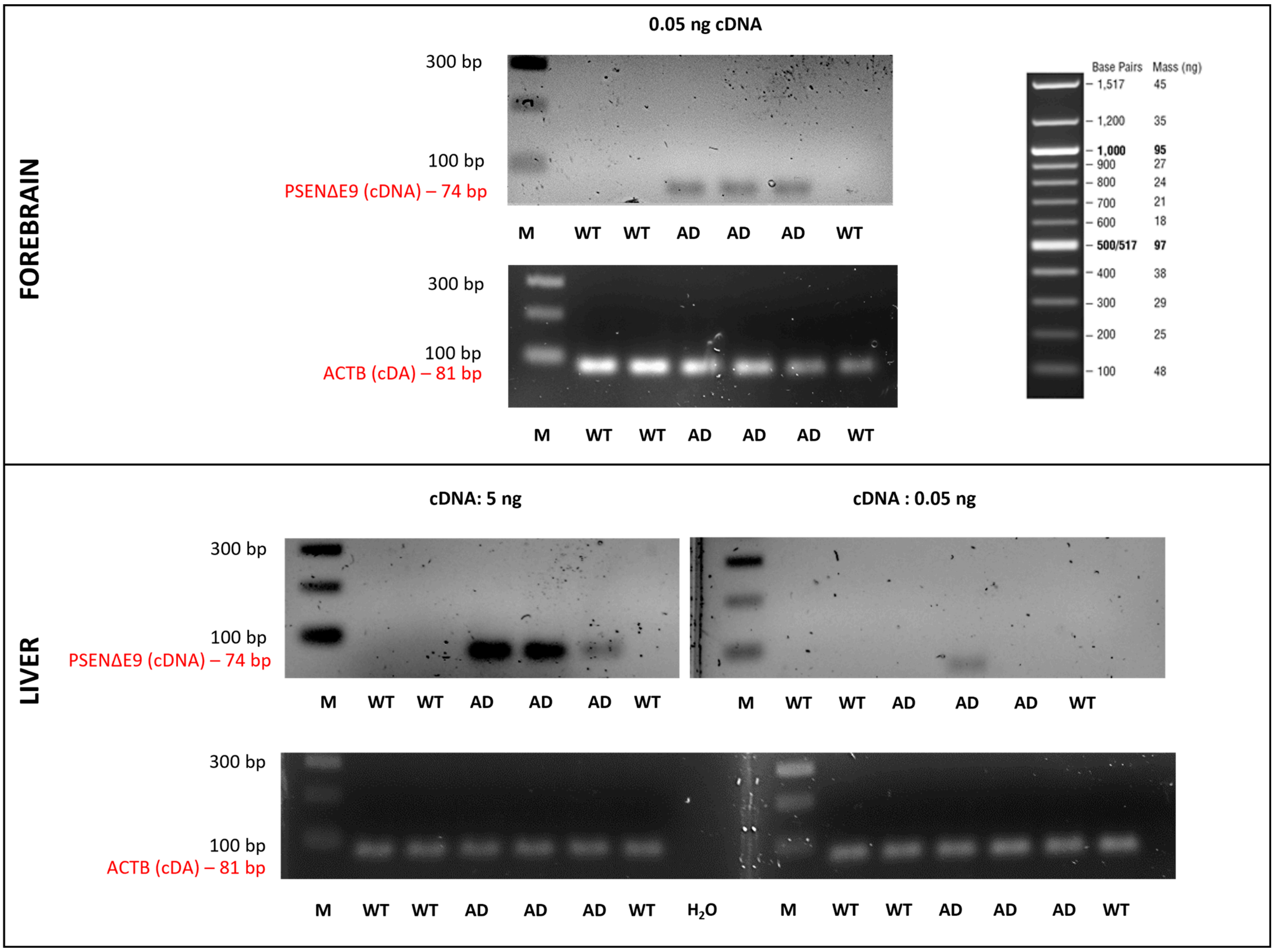

Figure S6. PSEN1ΔE9 expression in forebrain and liver of WT and APPswePS1ΔE9 (AD) mice. RNA was isolated from the forebrain and liver and converted to cDNA. After a PCR, the cDNA samples (including ethidium bromide 0.5 µg/mL) were run on a 2.5% agarose gel using 1x TAE electrophoresis buffer. The PCR (cDNA) samples and DNA (100 bp) molecular weight marker (Biolabs, #N3231S) were diluted in 10× loading buffer (0.25% bromphenol blue) and loaded into wells. Electrophoresis was conducted for 60 minutes at 100 V in 1x TAE buffer. After one hour, the stained DNA gel was put directly on a UV transilluminator and photographed.

Table S1. Comparison of APPswePS1ΔE9-related pathways and *H. elongata* lipid extract-affected pathways.

Canonical Pathways	APPswePS1ΔE9-Vehicle/ WT-Vehicle		APPswePS1ΔE9-*H. elongata*/ APPswePS1ΔE9-Vehicle		WT-*H. elongata*/ WT-Vehicle	
	−log(*p*-value)	Z-score	−log(*p*-value)	Z-score	−log(*p*-value)	Z-score
Calcium Signaling	2.99	-1.00	2.08	N/A	0.00	N/A
Acetylcholine Receptor Signaling Pathway *(Table S2)*	2.47	1.34	2.27	2.00	0.50	N/A
Synaptic Long Term Depression *(Table S2)*	1.71	1.00	1.46	N/A	0.00	N/A
Docosahexaenoic Acid (DHA) Signaling	1.39	2.00	1.89	2.00	0.36	N/A
Cachexia Signaling Pathway	1.91	1.63	0.83	N/A	1.66	0.82
Cardiac Hypertrophy Signaling (Enhanced)	4.60	2.53	0.51	N/A	0.00	N/A
G alpha (q) signalling events	4.46	2.65	0.00	N/A	0.00	N/A
S100 Family Signaling Pathway	4.32	2.67	0.52	1.00	0.00	N/A
Factors Promoting Cardiogenesis in Vertebrates	3.83	1.63	0.37	N/A	0.00	N/A
GPCR-Mediated Nutrient Sensing in Enteroendocrine Cells	3.43	1.34	0.46	N/A	0.00	N/A
G alpha (i) signalling events	3.40	0.38	0.22	N/A	0.00	N/A
Dopamine-DARPP32 Feedback in cAMP Signaling	3.38	1.00	0.84	N/A	0.00	N/A
Adrenergic Receptor Signaling Pathway (Enhanced)	3.21	1.63	0.29	N/A	0.00	N/A
DHCR24 Signaling Pathway	3.13	1.34	0.41	N/A	0.00	N/A
Endocannabinoid Neuronal Synapse Pathway	2.97	0.00	0.00	N/A	0.00	N/A
Dilated Cardiomyopathy Signaling Pathway	2.95	N/A	0.38	N/A	0.23	N/A
Role of NFAT in Cardiac Hypertrophy	2.95	0.82	0.26	N/A	0.00	N/A
Corticotropin Releasing Hormone Signaling	2.93	0.45	0.98	N/A	0.00	N/A
Opioid Signalling	2.91	1.00	0.00	N/A	0.00	N/A
CREB Signaling in Neurons	2.91	1.90	0.76	0.00	0.00	N/A
DAG and IP3 signaling	2.90	N/A	0.00	N/A	0.00	N/A
Estrogen Receptor Signaling	2.88	1.34	0.36	N/A	0.36	N/A
Class A/1 (Rhodopsin-like receptors)	2.88	1.13	0.00	N/A	0.25	N/A
Orexin Signaling Pathway	2.82	1.63	0.24	N/A	0.00	N/A
Dissolution of Fibrin Clot	2.70	N/A	0.00	N/A	0.00	N/A
Cardiac Hypertrophy Signaling	2.62	1.00	0.00	N/A	0.00	N/A
Cardiac Î²-adrenergic Signaling	2.61	N/A	0.00	N/A	0.00	N/A
Circadian Rhythm Signaling	2.56	N/A	1.14	N/A	0.00	N/A
Serotonin Receptor Signaling	2.51	2.12	0.30	N/A	0.00	N/A
GNRH Signaling	2.50	N/A	0.30	N/A	0.00	N/A
Opioid Signaling Pathway	2.47	0.82	0.00	N/A	0.00	N/A
Oxytocin Signaling Pathway	2.45	0.00	0.57	N/A	0.00	N/A
G-Protein Coupled Receptor Signaling	2.44	0.63	0.33	N/A	0.00	N/A
PCP (Planar Cell Polarity) Pathway	2.43	N/A	0.00	N/A	0.00	N/A
Oxytocin in Brain Signaling Pathway	2.42	-0.45	0.29	N/A	0.00	N/A
Human Embryonic Stem Cell Pluripotency	2.40	2.24	0.79	N/A	0.00	N/A
Electron transport, ATP synthesis, and heat production by uncoupling proteins	2.35	0.00	0.00	N/A	0.76	N/A
GABA Receptor Signaling	2.31	N/A	0.00	N/A	0.27	N/A
Axonal Guidance Signaling	2.29	N/A	0.56	N/A	0.24	N/A

White Adipose Tissue Browning Pathway	2.24	0.00	0.41	N/A	0.25	N/A
GABAergic Receptor Signaling Pathway (Enhanced)	2.23	0.00	0.00	N/A	0.71	N/A
Apelin Endothelial Signaling Pathway	2.20	0.00	0.40	N/A	0.25	N/A
Glutaminergic Receptor Signaling Pathway (Enhanced)	2.16	0.82	0.49	N/A	0.24	N/A
Neurovascular Coupling Signaling Pathway	2.14	1.34	1.29	N/A	0.81	N/A
Leptin Signaling in Obesity	2.14	N/A	0.00	N/A	0.00	N/A
Cellular Effects of Sildenafil (Viagra)	2.11	N/A	0.38	N/A	0.00	N/A
WNT ligand biogenesis and trafficking	2.10	N/A	0.00	N/A	0.00	N/A
Effects of PIP2 hydrolysis	2.07	N/A	1.03	N/A	0.00	N/A
Cardiomyocyte Differentiation via BMP Receptors	2.07	N/A	0.00	N/A	0.00	N/A
Transcriptional Regulatory Network in Embryonic Stem Cells	1.98	1.00	0.35	N/A	0.00	N/A
Glioblastoma Multiforme Signaling	1.92	2.00	0.00	N/A	0.00	N/A
Class B/2 (Secretin family receptors)	1.87	N/A	0.53	N/A	0.00	N/A
Insulin Secretion Signaling Pathway	1.85	N/A	0.58	N/A	0.00	N/A
Striated Muscle Contraction	1.83	N/A	0.91	N/A	0.00	N/A
NGF-stimulated transcription	1.76	N/A	0.00	N/A	0.00	N/A
Formation of Fibrin Clot (Clotting Cascade)	1.76	N/A	0.00	N/A	0.00	N/A
PPARÎ±/RXRÎ± Activation	1.74	N/A	0.00	N/A	0.00	N/A
Pulmonary Healing Signaling Pathway	1.70	2.00	0.29	N/A	0.00	N/A
Gustation Pathway	1.68	1.00	0.28	N/A	0.48	N/A
Autism Signaling Pathway	1.66	1.34	0.00	N/A	0.00	N/A
Hepatic Fibrosis Signaling Pathway	1.64	2.00	0.35	N/A	0.00	N/A
tRNA Splicing	1.63	N/A	0.00	N/A	0.62	N/A
Role of OCT4 in Mammalian Embryonic Stem Cell Pluripotency	1.63	N/A	0.00	N/A	0.00	N/A
Cholecystokinin/Gastrin-mediated Signaling	1.62	N/A	0.46	N/A	0.00	N/A
Nitric Oxide Signaling in the Cardiovascular System	1.61	N/A	1.16	N/A	0.29	N/A
Renin-Angiotensin Signaling	1.60	N/A	0.45	N/A	0.80	N/A
Galactose Degradation I (Leloir Pathway)	1.59	N/A	0.00	N/A	0.00	N/A
G alpha (z) signalling events	1.59	N/A	0.80	N/A	0.00	N/A
nNOS Signaling in Skeletal Muscle Cells	1.59	N/A	0.00	N/A	0.00	N/A
LXR/RXR Activation	1.58	N/A	0.00	N/A	0.00	N/A
Role of NANOG in Mammalian Embryonic Stem Cell Pluripotency	1.57	N/A	0.00	N/A	0.00	N/A
ROBO SLIT Signaling Pathway	1.55	N/A	0.00	N/A	0.28	N/A
Phagosome Formation	1.55	1.41	0.97	1.34	0.00	N/A
Transcriptional regulation of granulopoiesis	1.54	N/A	0.00	N/A	0.00	N/A
G Beta Gamma Signaling	1.53	N/A	0.43	N/A	0.00	N/A
Thrombin Signaling	1.53	1.00	0.25	N/A	0.00	N/A
Role of Macrophages, Fibroblasts and Endothelial Cells in Rheumatoid Arthritis	1.53	N/A	0.47	N/A	0.00	N/A
Serotonin and Melatonin Biosynthesis	1.51	N/A	0.00	N/A	0.00	N/A
P2Y Purigenic Receptor Signaling Pathway	1.50	N/A	0.42	N/A	0.00	N/A
GÎ±12/13 Signaling	1.50	N/A	0.00	N/A	0.00	N/A
Mitochondrial Dysfunction	1.48	1.34	0.00	N/A	0.84	0.00
Molecular Mechanisms of Cancer	1.47	2.33	0.71	1.34	0.00	N/A
cAMP-mediated signaling	1.46	2.00	0.68	N/A	0.39	N/A
Breast Cancer Regulation by Stathmin1	1.46	1.89	0.78	1.00	0.00	N/A
TYSND1 cleaves peroxisomal proteins	1.45	N/A	0.00	N/A	0.00	N/A
GABA receptor activation	1.41	N/A	0.00	N/A	0.53	N/A

G alpha (s) signalling events	1.40	N/A	0.39	N/A	0.68	N/A
Pancreatic Secretion Signaling Pathway	1.40	2.00	0.65	N/A	0.00	N/A
rRNA modification in the mitochondrion	1.39	N/A	0.00	N/A	0.00	N/A
Cytoprotection by HMOX1	1.39	N/A	0.00	N/A	0.00	N/A
BBSome Signaling Pathway	1.38	1.63	0.59	N/A	0.00	N/A
WNT/SHH Axonal Guidance Signaling Pathway	1.37	N/A	0.38	N/A	0.23	N/A
Type II Diabetes Mellitus Signaling	1.34	N/A	0.98	N/A	0.00	N/A
Sucrose Degradation V (Mammalian)	1.34	N/A	0.00	N/A	1.29	N/A
Relaxin Signaling	1.33	N/A	0.37	N/A	0.22	N/A
Sensory processing of sound by inner hair cells of the cochlea	1.30	N/A	0.00	N/A	0.48	N/A
eNOS Signaling	0.71	N/A	2.61	2.00	1.87	-1.00
Cellular response to heat stress	0.00	N/A	2.28	N/A	2.59	1.00
ID1 Signaling Pathway	1.06	N/A	1.44	N/A	0.48	N/A
Salvage Pathways of Pyrimidine Ribonucleotides	1.05	N/A	1.32	N/A	0.00	N/A
Oxytocin in Spinal Neurons Signaling Pathway	0.78	N/A	2.15	N/A	0.73	N/A
nNOS Signaling in Neurons	0.66	N/A	1.90	N/A	0.00	N/A
Synaptogenesis Signaling Pathway *(Table S2)*	0.65	N/A	1.57	2.00	0.00	N/A
Pyridoxal 5'-phosphate Salvage Pathway	0.54	N/A	1.64	N/A	0.00	N/A
Macropinocytosis Signaling	0.49	N/A	1.51	N/A	0.00	N/A
Prolactin Signaling	0.41	N/A	1.34	N/A	0.37	N/A
SNARE Signaling Pathway	0.30	N/A	1.88	N/A	0.26	N/A
Acetylcholine binding and downstream events	0.00	N/A	4.80	N/A	0.00	N/A
Peroxisomal lipid metabolism	0.00	N/A	2.55	N/A	0.00	N/A
Regulation of Cellular Mechanics by Calpain Protease	0.00	N/A	1.38	N/A	0.00	N/A
NAD Phosphorylation and Dephosphorylation	0.00	N/A	1.34	N/A	0.00	N/A
Regulation of TP53 Activity through Association with Co-factors	0.00	N/A	1.31	N/A	0.00	N/A
Neuroprotective Role of THOP1 in Alzheimer's Disease	0.88	N/A	0.00	N/A	2.23	N/A
Endocannabinoid Cancer Inhibition Pathway	0.75	N/A	0.00	N/A	1.95	0.00
Huntington's Disease Signaling	0.74	N/A	0.56	N/A	3.55	N/A
TP53 Regulates Transcription of Cell Death Genes	0.69	N/A	0.00	N/A	1.56	N/A
Regulation of mRNA stability by proteins that bind AU-rich elements	0.69	N/A	0.00	N/A	1.56	N/A
Aldosterone Signaling in Epithelial Cells	0.65	N/A	0.89	N/A	1.72	N/A
Collagen biosynthesis and modifying enzymes	0.53	N/A	0.00	N/A	2.14	N/A
Integrin cell surface interactions	0.45	N/A	0.58	N/A	1.86	N/A
Immunogenic Cell Death Signaling Pathway	0.43	N/A	0.00	N/A	1.79	N/A
Death Receptor Signaling	0.41	N/A	0.00	N/A	1.72	N/A
Apoptosis Signaling	0.00	N/A	1.27	N/A	1.63	N/A
BAG2 Signaling Pathway	0.00	N/A	0.58	N/A	1.86	N/A
PIP3 activates AKT signaling	0.00	N/A	0.40	N/A	1.30	N/A
Serine biosynthesis	0.00	N/A	0.00	N/A	3.03	N/A
Response of EIF2AK1 (HRI) to heme deficiency	0.00	N/A	0.00	N/A	2.47	N/A
Endoplasmic Reticulum Stress Pathway	0.00	N/A	0.00	N/A	2.17	N/A
TCA Cycle II (Eukaryotic)	0.00	N/A	0.00	N/A	2.10	N/A
Role of PKR in Interferon Induction and Antiviral Response	0.00	N/A	0.00	N/A	2.07	0.00
Tumoricidal Function of Hepatic Natural Killer Cells	0.00	N/A	0.00	N/A	2.06	N/A
Unfolded protein response	0.00	N/A	0.00	N/A	1.79	N/A
Serine Biosynthesis	0.00	N/A	0.00	N/A	1.76	N/A

Coenzyme A Biosynthesis	0.00	N/A	0.00	N/A	1.76	N/A
Pyrophosphate hydrolysis	0.00	N/A	0.00	N/A	1.76	N/A
Melanin biosynthesis	0.00	N/A	0.00	N/A	1.76	N/A
TWEAK Signaling	0.00	N/A	0.00	N/A	1.70	N/A
Glucocorticoid Receptor Signaling	0.00	N/A	0.00	N/A	1.66	N/A
Protein Ubiquitination Pathway	0.00	N/A	0.00	N/A	1.64	N/A
Methylmalonyl Pathway	0.00	N/A	0.00	N/A	1.63	N/A
PI3K Cascade	0.00	N/A	0.00	N/A	1.56	N/A
Superpathway of Serine and Glycine Biosynthesis I	0.00	N/A	0.00	N/A	1.54	N/A
2-oxobutanoate Degradation I	0.00	N/A	0.00	N/A	1.54	N/A
Choline catabolism	0.00	N/A	0.00	N/A	1.54	N/A
DNA damage-induced 14-3-3Ïƒ Signaling	0.00	N/A	0.00	N/A	1.52	N/A
Mitotic G2-G2/M phases	0.00	N/A	0.00	N/A	1.52	0.00
Cilium Assembly	0.00	N/A	0.00	N/A	1.49	2.00
Fructose metabolism	0.00	N/A	0.00	N/A	1.46	N/A
Glycine Cleavage Complex	0.00	N/A	0.00	N/A	1.46	N/A
Synthesis of Lipoxins (LX)	0.00	N/A	0.00	N/A	1.46	N/A
TNFR1 Signaling	0.00	N/A	0.00	N/A	1.44	N/A
Aspartate Degradation II	0.00	N/A	0.00	N/A	1.39	N/A
HSP90 chaperone cycle for steroid hormone receptors in the presence of ligand	0.00	N/A	0.00	N/A	1.38	N/A
Intrinsic Pathway for Apoptosis	0.00	N/A	0.00	N/A	1.38	N/A
The citric acid (TCA) cycle and respiratory electron transport	0.00	N/A	0.00	N/A	1.38	N/A
Polyamine Regulation in Colon Cancer	0.00	N/A	0.00	N/A	1.33	N/A
Metabolism of water-soluble vitamins and cofactors	0.00	N/A	0.00	N/A	1.33	N/A

Table S2. Ratios of cholesterol precursors, cholesterol metabolites and phytosterols to cholesterol concentrations.

	WT	APPswePS1ΔE9		
**Cerebellum**	Vehicle	Vehicle	*H. elongata*	*S. fusiforme* SCF
**Cholesterol precursors**				
Lanosterol/cholesterol	1.00 ± 0.18	**0.75 ± 0.06 ###**	0.73 ± 0.11	0.75 ± 0.06
Desmosterol/cholesterol	1.00 ± 0.15	0.97 ± 0.13	**3.40 ± 0.58 *****	**1.64 ± 0.23 *****
Lathosterol/cholesterol $	1.00 ± 0.21	0.89 ± 0.14	**0.58 ± 0.09 *****	**0.71 ± 0.09 ***
**Cholesterol metabolites**				
24-OHC/cholesterol	1.00 ± 0.29	**0.79 ± 0.08 #**	0.84 ± 0.10	0.78 ± 0.11
27-OHC/cholesterol $	1.00 ± 0.28	1.08 ± 0.31	1.09 ± 0.43	1.34 ± 0.32
7α-OHC/cholesterol	1.00 ± 0.38	**1.58 ± 0.26 ###**	**2.02 ± 0.43 ***	1.69 ± 0.63
Cholestanol/cholesterol $	1.00 ± 0.26	0.97 ± 0.47	0.70 ± 0.33	0.51 ± 0.34
**Liver**				
	**WT**	**APPswePS1ΔE9**		
	Vehicle	Vehicle	*H. elongata*	*S. fusiforme* SCF
**Cholesterol precursors**				
Lanosterol/cholesterol	1.00 ± 0.36	0.99 ± 0.30	**2.46 ± 0.78 *****	**2.23 ± 0.55 *****
Desmosterol/cholesterol	1.00 ± 0.16	**1.18 ± 0.19 #**	**108.17 ± 23.26 *****	**50.16 ± 14.42 *****
Lathosterol/cholesterol	1.00 ± 0.21	0.91 ± 0.17	**1.23 ± 0.45 ***	**1.23 ± 0.15 ***
**Cholesterol metabolites**				
24-OHC/cholesterol	1.00 ± 0.18	**2.05 ± 0.42 ###**	**3.11 ± 0.67 *****	2.46 ± 0.59
27-OHC/cholesterol	1.00 ± 0.23	1.16 ± 0.21	1.07 ± 0.20	1.25 ± 0.18
7α-OHC/cholesterol $	1.00 ± 0.33	**4.39 ± 1.64 ###**	**9.91 ± 4.58 ****	5.12 ± 3.87
Cholestanol/cholesterol	1.00 ± 0.14	0.91 ± 0.12	**0.71 ± 0.15 ****	**0.75 ± 0.14 ***
**Phytosterols**				
Saringosterol/cholesterol $	1.00 ± 0.08	1.02 ± 0.09	**15.08 ± 5.42 ****	**38.36 ± 11.18 *****
Fucosterol/cholesterol $	1.00 ± 0.11	0.92 ± 0.09	**8.31 ± 0.1.46 ****	**12.66 ± 3.13 *****
Campesterol/cholesterol	1.00 ± 0.18	1.05 ± 0.19	**0.10 ± 0.02 *****	**0.13 ± 0.04 *****
Campestanol/cholesterol	1.00 ± 0.18	1.03 ± 0.20	**0.36 ± 0.04 *****	**0.48 ± 0.10 *****
Sitosterol/cholesterol $	1.00 ± 0.19	1.00 ± 0.18	**0.18 ± 0.02 *****	**0.23 ± 0.05 ****
Sitostanol/cholesterol $	1.00 ± 0.16	1.01 ± 0.12	**0.70 ± 0.06 *****	**0.76 ± 0.15 ****
Avenasterol/cholesterol	1.00 ± 0.18	1.04 ± 0.20	**0.31 ± 0.04 *****	**0.41 ± 0.08 *****
Brassicasterol/cholesterol $	1.00 ± 0.12	1.05 ± 0.12	**0.53 ± 0.07 *****	**0.48 ± 0.09 *****
Stigmasterol/cholesterol $	1.00 ± 0.12	0.94 ± 0.11	**0.74 ± 0.08 ****	**0.68 ± 0.12 *****
**Serum**				
	**WT**	**APPswePS1ΔE9**		
	Vehicle	Vehicle	*H. elongata*	*S. fusiforme* SCF
**Cholesterol precursors**				
Lanosterol/cholesterol $	1.00 ± 0.23	1.13 ± 0.28	**2.51 ± 1.21 *****	1.79 ± 0.23 *
Desmosterol/cholesterol	1.00 ± 0.13	1.09 ± 0.22	**120.76 ± 30.90 *****	**44.30 ± 6.90 *****
Lathosterol/cholesterol	1.00 ± 0.21	1.00 ± 0.19	**0.73 ± 0.17 ****	**0.74 ± 0.14 ****
**Cholesterol metabolites**				
24-OHC/cholesterol $	1.00 ± 0.15	1.14 ± 0.18	1.24 ± 0.29	1.09 ± 0.12
27-OHC/cholesterol $	1.00 ± 0.12	1.02 ± 0.12	**0.83 ± 0.15**	**1.37 ± 0.25 ***
7α-OHC/cholesterol $	1.00 ± 0.32	1.12 ± 0.28	1.03 ± 0.17	**0.88 ± 0.06 ****
Cholestanol/cholesterol	1.00 ± 0.13	1.10 ± 0.13	**0.72 ± 0.10 *****	**0.74 ± 0.14 *****
**Phytosterols**				
Saringosterol/cholesterol	1.00 ± 0.11	**0.80 ± 0.11 ###**	**9.61 ± 2.97 *****	**20.91 ± 4.02 *****
Fucosterol/cholesterol $	1.00 ± 0.07	**0.68 ± 0.07 ###**	**8.51 ± 1.21 ****	**11.60 ± 2.93*****
Campesterol/cholesterol	1.00 ± 0.12	**1.22 ± 0.17 ##**	**0.12 ± 0.03 *****	**0.14 ± 0.05 *****
Campestanol/cholesterol $	1.00 ± 0.14	**1.41 ± 0.24 ###**	**0.26 ± 0.03 *****	**0.32 ± 0.08 ****
Sitosterol/cholesterol	1.00 ± 0.10	**1.33 ± 0.22 ###**	**0.17 ± 0.03 *****	**0.22 ± 0.06 *****
Sitostanol/cholesterol $	1.00 ± 0.08	**1.47 ± 0.17 ###**	**0.73 ± 0.12 *****	**0.67 ± 0.13 *****
Avenasterol/cholesterol $	1.00 ± 0.10	**0.75 ± 0.12 ###**	**0.29 ± 0.04 *****	**0.33 ± 0.06 ****
Brassicasterol/cholesterol $	1.00 ± 0.08	**1.15 ± 0.18 #**	**0.00 ± 0.00 *****	**0.00 ± 0.00 *****
Stigmasterol/cholesterol	1.00 ± 0.08	**1.18 ± 0.14 ##**	**0.77 ± 0.16 *****	**0.64 ± 0.10 *****

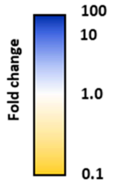

The data are presented as fold change relative to WT-vehicle and are presented as mean ± SD (n = 9–13 per group). Differences between vehicle-treated APPswePS1ΔE9 and WT mice were analyzed with an unpaired t-test or Mann Whitney U test (indicated with $) (# $p \leq 0.05$; ## $p \leq 0.01$; ### $p \leq 0.001$); Treatment effects in APPswePS1ΔE9 mice were analyzed with one-way ANOVA (with Dunnett's multiple comparisons test) or Kruskal-Wallis test (with Dunn's multiple comparisons test) (indicated with $) (* $p \leq 0.05$, ** $p \leq 0.01$, *** $p \leq 0.001$).
